# Recent Advances in the Synthesis and Biomedical Applications of Heterocyclic NO-Donors

**DOI:** 10.3390/molecules26185705

**Published:** 2021-09-21

**Authors:** Leonid L. Fershtat, Egor S. Zhilin

**Affiliations:** Laboratory of Nitrogen Compounds, N.D. Zelinsky Institute of Organic Chemistry, Russian Academy of Sciences, Leninsky Prosp., 47, 119991 Moscow, Russia; es.zhilin@gmail.com

**Keywords:** nitric oxide, heterocycles, sydnone imines, furoxans, pyridazine dioxides, azasydnones, pharmacologically-active compounds

## Abstract

Nitric oxide (NO) is a key signaling molecule that acts in various physiological processes such as cellular metabolism, vasodilation and transmission of nerve impulses. A wide number of vascular diseases as well as various immune and neurodegenerative disorders were found to be directly associated with a disruption of NO production in living organisms. These issues justify a constant search of novel NO-donors with improved pharmacokinetic profiles and prolonged action. In a series of known structural classes capable of NO release, heterocyclic NO-donors are of special importance due to their increased hydrolytic stability and low toxicity. It is no wonder that synthetic and biochemical investigations of heterocyclic NO-donors have emerged significantly in recent years. In this review, we summarized recent advances in the synthesis, reactivity and biomedical applications of promising heterocyclic NO-donors (furoxans, sydnone imines, pyridazine dioxides, azasydnones). The synthetic potential of each heterocyclic system along with biochemical mechanisms of action are emphasized.

## 1. Introduction

Nitric oxide (NO) (also known as an endothelium-derived relaxing factor) is an endogenous inorganic soluble gas produced in mammalians from L-arginine and molecular oxygen by the enzyme nitric oxide synthase (NOS) [1,2]. NO is one of the most versatile molecules in animal and human biology with diverse roles in both physiology and pathophysiology. NO exhibits vasodilating properties with anti-smooth muscle cell activity [3], inhibits platelet adhesion and aggregation [4] and has other anti-inflammatory properties [5]. In addition, NO is capable of neurotransmittance and neuromodulation due to its involvement in cerebral blood flow auto- and chemoregulation [6].

In 1998, Furchgott, Ignarro and Murad received a Nobel Prize in Physiology or Medicine “for their discoveries concerning nitric oxide as a signaling molecule in the cardiovascular system” [7,8,9]. Since then, researches on the biochemical roles of NO have grown rapidly. It was found that NO plays also an important role in a number of pathophysiological diseases, such as arthritis, atherosclerosis, cancer, diabetes and various degenerative neuronal diseases [10,11,12]. Different release patterns of NO by different NO donors can modulate angiogenesis differentially: it was shown that while the short term NO donors are primarily active to initiate the angiogenesis by inducing cellular migration and ring formation, long term NO donors define later stages of angiogenesis such as vessel maturation and neovascularization [13]. NO donor or overexpression of endothelial NO synthase fused to a green fluorescent protein (eNOS-GFP) has a protective effect against hypoxia-induced cellular deadhesion and greatly improves the redox balance by inhibiting the oxidative stress [14]. Ectopic release of NO stimulates the protection of the endothelium leaky and improves actin dynamics under hypoxia milieu in chick embryo extravascular models [15]. At the same time, overexpression of exogenous NO levels in chicken embryos may increase the cell migration and cell proliferation on the right-hand side of the heart resulting in the *situs inversus* which is referred to as a congenital condition comprising of the reversion of the major visceral organs from their normal positions [16]. Recently, regulation of nitrosative and oxidative stresses by NO and its influence on lung diseases and cardiogenesis were thoroughly reviewed [17,18]. No wonder, the discovery of such crucial and indispensable biochemical patterns of NO stimulated a search of prodrug candidates capable of NO release under physiological conditions [19,20,21,22,23]. Overall, the creation of efficient methodologies for the construction of novel NO-donor heterocyclic and acyclic systems became one of the rapidly developing fields in organic and medicinal chemistry.

Glyceryl trinitrate (GTN) is a well-known, approved and inexpensive NO-donor, which lowers blood pressure and increases heart rate. However, GTN suffers from various side effects, such as headache, difficult or labored breathing, dizziness, and also may induce nitrate tolerance upon continuous exposure [24]. A similar pharmacological profile matches other organic nitrate-based pharmacologically active substances (e.g., isosorbide dinitrate) [25], although their levels of NO release are quite different [26]. Aside from organic nitrates, other nitrogen–oxygen acyclic species were reported as NO-donors: *C*- or *N*-nitroso compounds, nitrosothiols, oximes, hydroxylamines, hydroxyurea and metal-nitrosyl complexes (sodium nitroprusside) [22]. However, in recent years, heterocyclic NO-donors emerged with special attention due to their hydrolytic stability, safer storage and absence of tolerance [22,23,27]. The progress made in the design, synthesis and biochemistry of heterocyclic NO-donors in the last decade unveiled an application potential of such organic nitrogen–oxygen molecular systems in medicinal chemistry and drug design.

**Figure 1:**
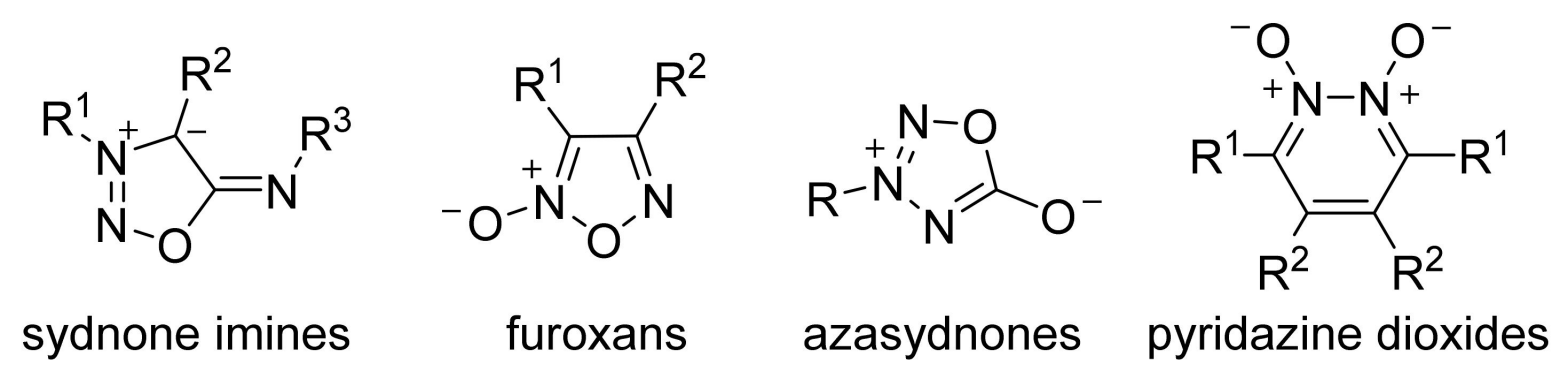
Structures of heterocyclic NO-donors presented in this review.

Therefore, in this review, we summarized recent advances in the synthesis and reactivity of structurally diverse NO-donors incorporating nitrogen–oxygen-enriched heterocyclic scaffold: sydnone imines, furoxans, azasydnones and pyridazine dioxides (Figure 1). These heterocyclic subclasses were chosen due to an increased number of researches on their synthesis, functionalization and properties. Main trends in synthetic methodologies for each type of heterocycle are presented. NO-releasing properties, pharmacological activity and other biomedical applications along with an analysis of structure–property relationships are also considered.

## 2. Sydnone Imines

**Figure 2:**
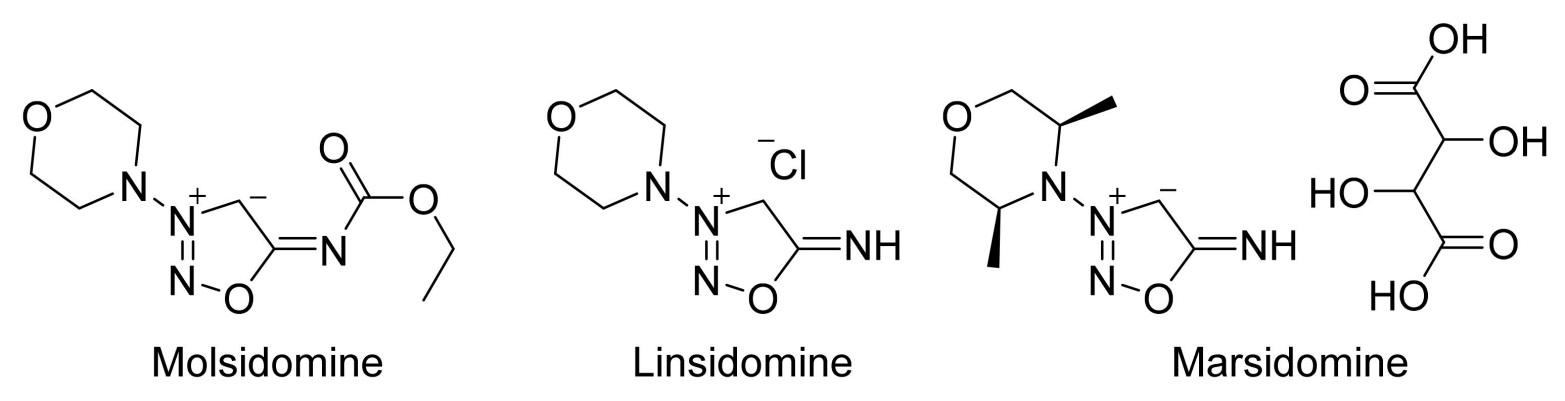
Structures of sydnone imine-based pharmaceuticals.

The 1,2,3-oxadiazol-3-ium-5-aminides, also known as sydnone imines, are referred to as mesoionic heterocycles and constitute a considerable part of exogenous nitric oxide donors [28]. Due to the ability of NO release, iminosydnones are of great interest in the development of novel pharmaceutical ingredients. The most prominent of them, for example, are Linsidomine, Molsidomine and Marsidomine (Figure 2) which have improved pharmacological profiles due to the presence of the saturated nitrogen heterocyclic subunit linked to the sydnone imine scaffold via NN bond.

**Scheme 1:**
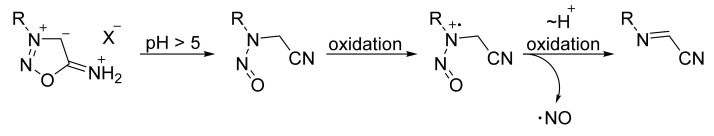
Mechanism of NO release from sydnone imines.

At physiological and a more alkaline pH, sydnone imines undergo rapid nonenzymatic hydrolysis to form the ring-opened *N*-nitrosamine, which is stable at pH 7.4 under anaerobic conditions protected from light. Traces of oxygen promote oxidative conversion to a cation radical intermediate which releases NO (Scheme 1) [22]. Interestingly, irradiation with visible light can remarkably enhance the oxygen-dependent NO release from sydnone imines [29].

**Scheme 2:**
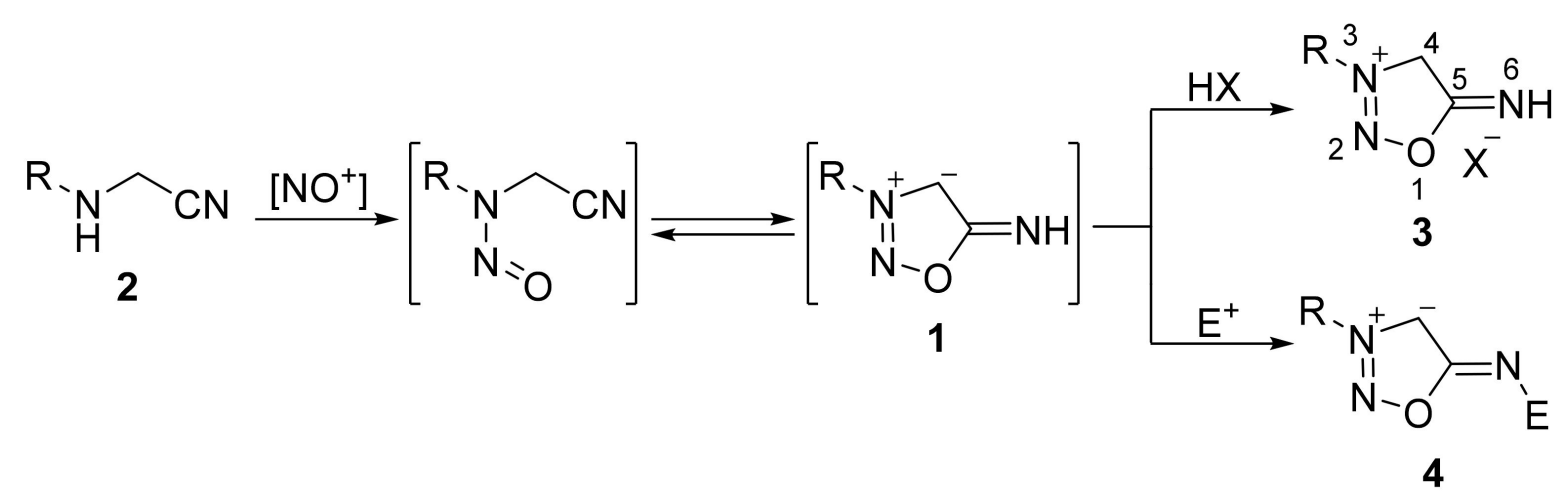
Assembly of the sydnone imine ring.

N^3^-Substituted sydnone imines **1** can be synthesized according to a standard method [30,31] via nitrosation of the corresponding amino nitriles **2** followed by cyclization to the target mesoionic heterocycle (Scheme 2). However, the cyclization is reversible and possible ring cleavage occurs through several degradation pathways depending on the solvent and pH of the reaction media. Therefore, to obtain stable sydnone imines suitable for storage and applications, they are usually converted to salts **3** or exo-*N*-substituted derivatives **4** [32].

**Scheme 3:**
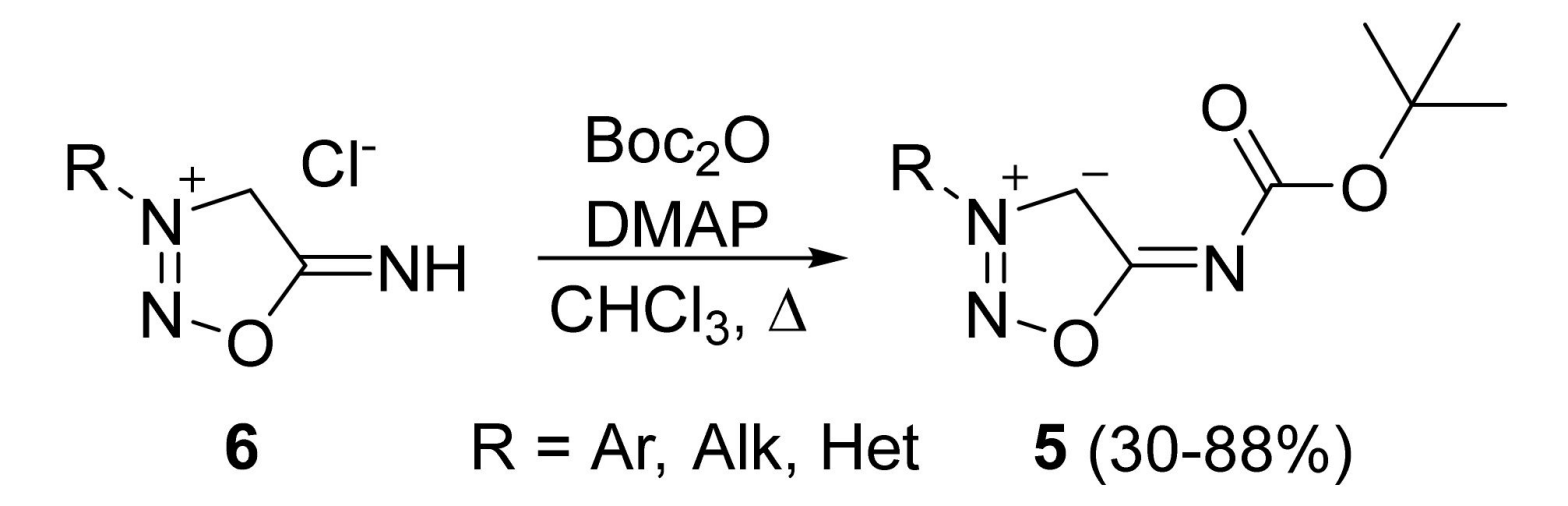
Interaction of sydnone imine hydrochlorides with Boc_2_O.

In general, there are two reactive centers in the sydnone imine cycle–C^4^ and N^6^ positions (for clarity, the numbering of atoms is shown in Scheme 2), both of which represent nucleophilic properties. However, the N^6^ atom is much more nucleophilic, which allows for performing selective functionalization of the sydnone imine scaffold through a preliminary transformation of free iminosydnone base into N^6^-derived substances followed by C^4^ modification. Reactions of sydnone imines at the N^6^ position afford a wide diversity of variously substituted amides. The synthesis of N^6^-derived sydnone imines is usually based on an interaction of the corresponding iminosydnone with various carbonyl electrophiles. For example, N^6^-*tert*-butoxycarbonylsydnone imines **5** were synthesized by acylation of sydnone imine hydrochlorides **6** with Boc_2_O in the presence of a catalytic amount of 4-dimethylaminopyridine (DMAP) in mild conditions (Scheme 3) [33].

**Scheme 4:**
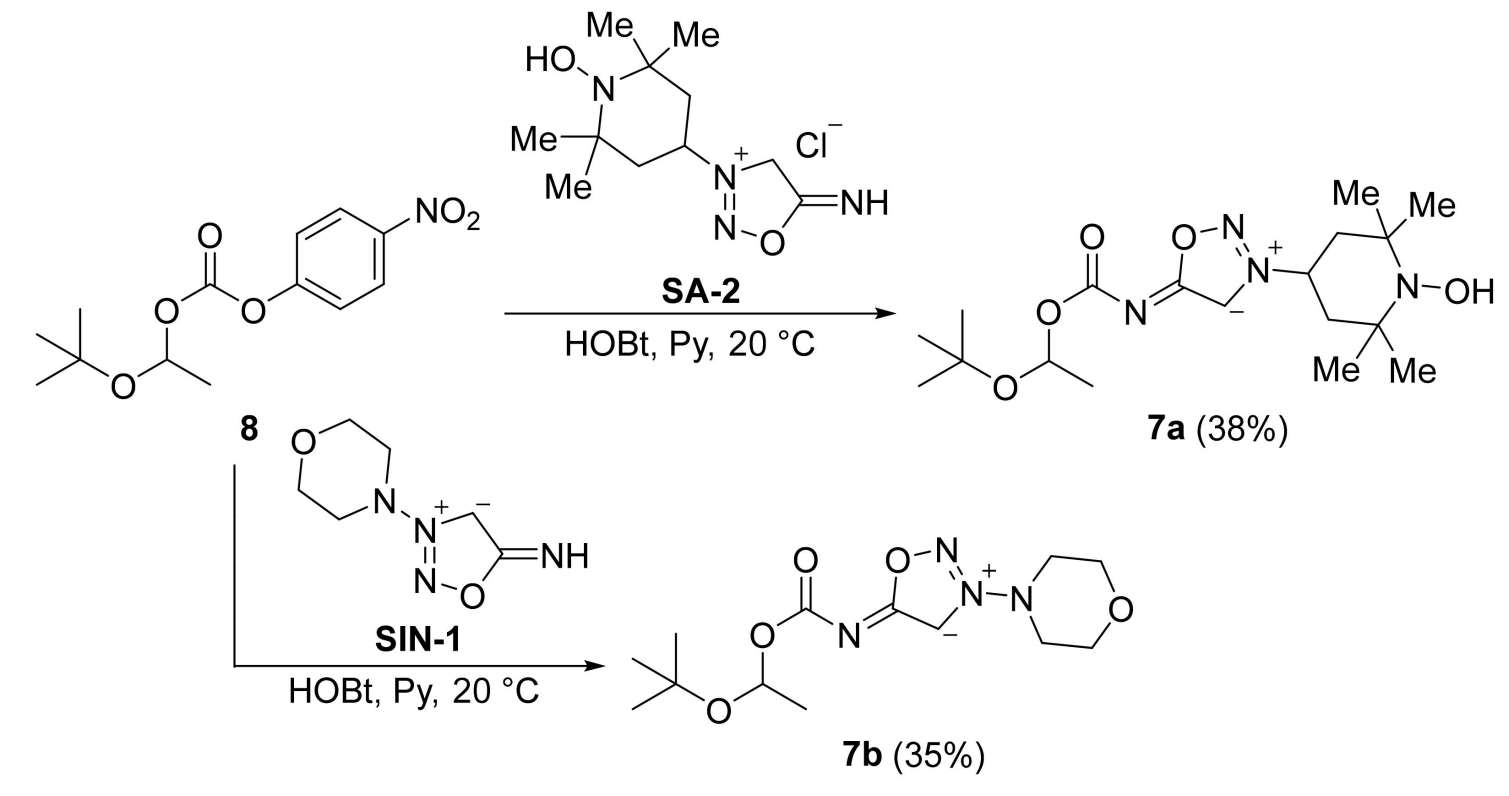
Acylation of sydnone imines with pivaloxy anhydride **8**.

N^6^-Carbamoyl sydnone imines **7a,b** bearing an acyloxy alkyl carbamate motif were reported [34] as ocular prodrugs and were prepared in moderate yields by an interaction of pharmaceutically relevant substances **SIN-1** or **SA-2** with pivaloxy anhydride **8** in the presence of hydroxybenzotriazole and pyridine (Scheme 4).

**Scheme 5:**
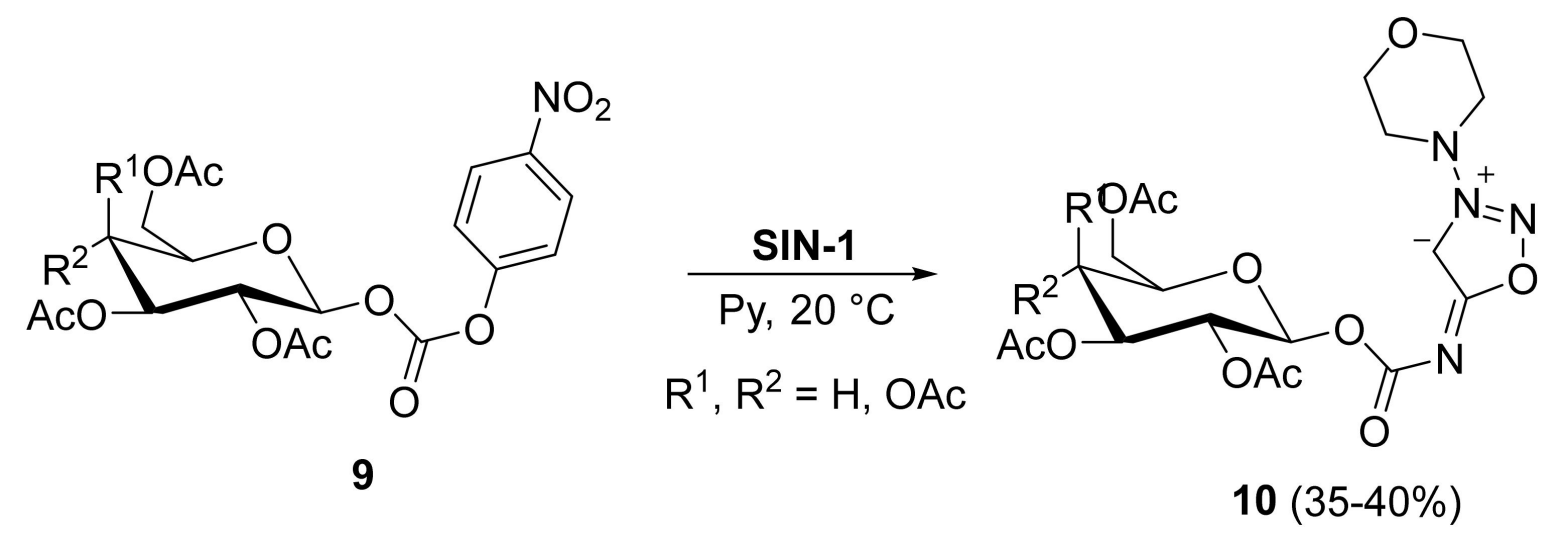
Synthesis of sydnone imine glycosyl carbamates **10**.

The 4-nitrophenyl carbonate derivatives **9** may also be used as suitable acylating agents to incorporate carbohydrate motifs onto the sydnone imine scaffold. Using this method, a few examples of sydnone imine glycosyl carbamates **10** representing a new class of glycosidase-dependent NO donors were prepared (Scheme 5) [35].

**Scheme 6:**
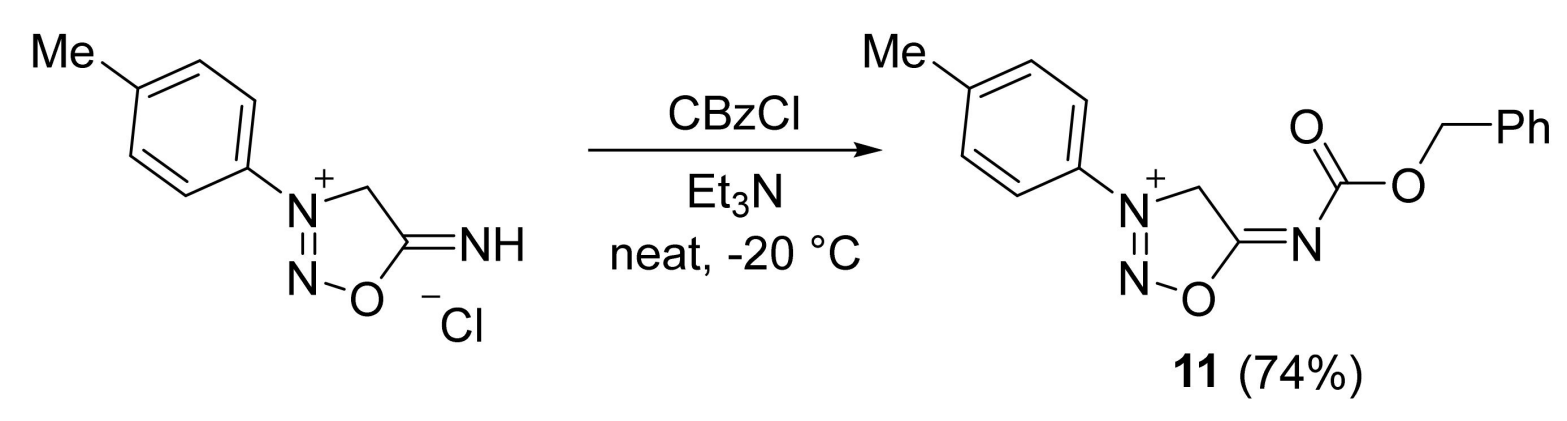
Acylation of sydnone imines with CBzCl.

Iminosydnones **11** bearing an oxycarbonyl moiety can also be synthesized using chloroformates instead of anhydrides to perform an acylation of the imine function (Scheme 6) [36].

**Scheme 7:**
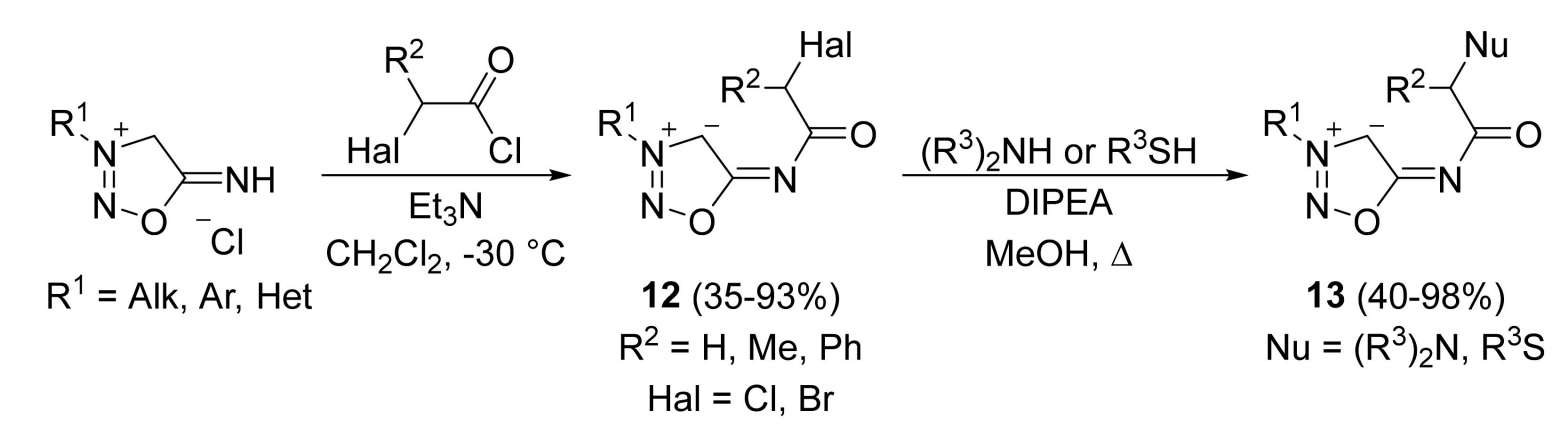
Synthesis of *α*-amino- and *α*-thio-substituted acyl sydnone imines.

A mild method for the synthesis of N^6^-*α*-haloacyl substituted sydnone imines **12** based on a treatment of parent mesoionic heterocycles with *α*-haloacyl chlorides was developed [37]. Compounds **12** were shown to be convenient intermediates for the preparation of a large variety of N^6^-*α*-amino- and N^6^-*α*-thio-substituted acyl sydnone imines **13** (Scheme 7).

**Scheme 8:**
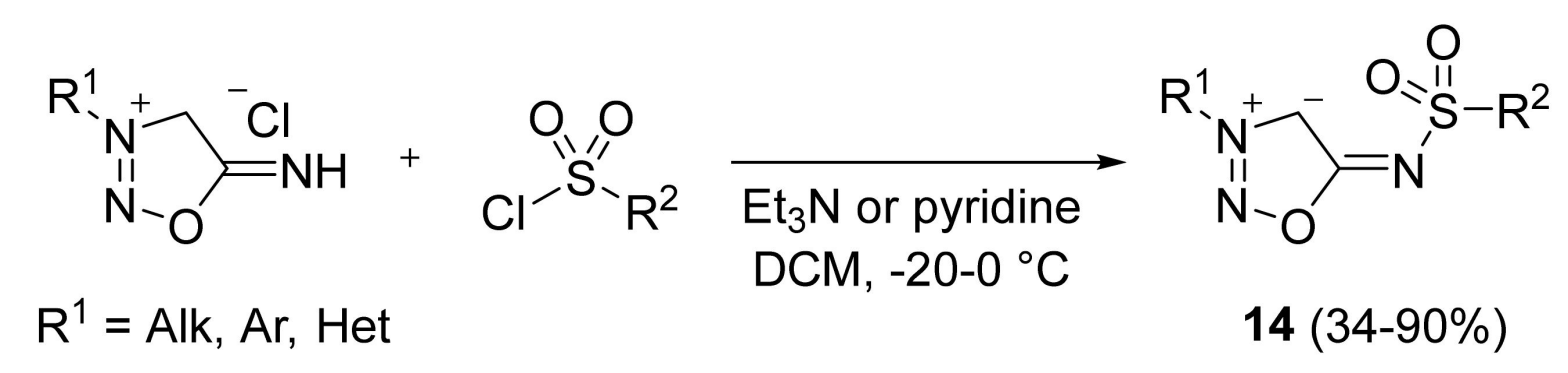
Synthesis of N^6^-sulfonyl iminosydnones **14**.

An interaction of sydnone imine hydrochlorides with sulfonyl chlorides in basic media afforded N^6^-sulfonyl iminosydnones **14** which have a great potential in bioorthogonal click-and-release methodology (Scheme 8) [38,39].

**Scheme 9:**
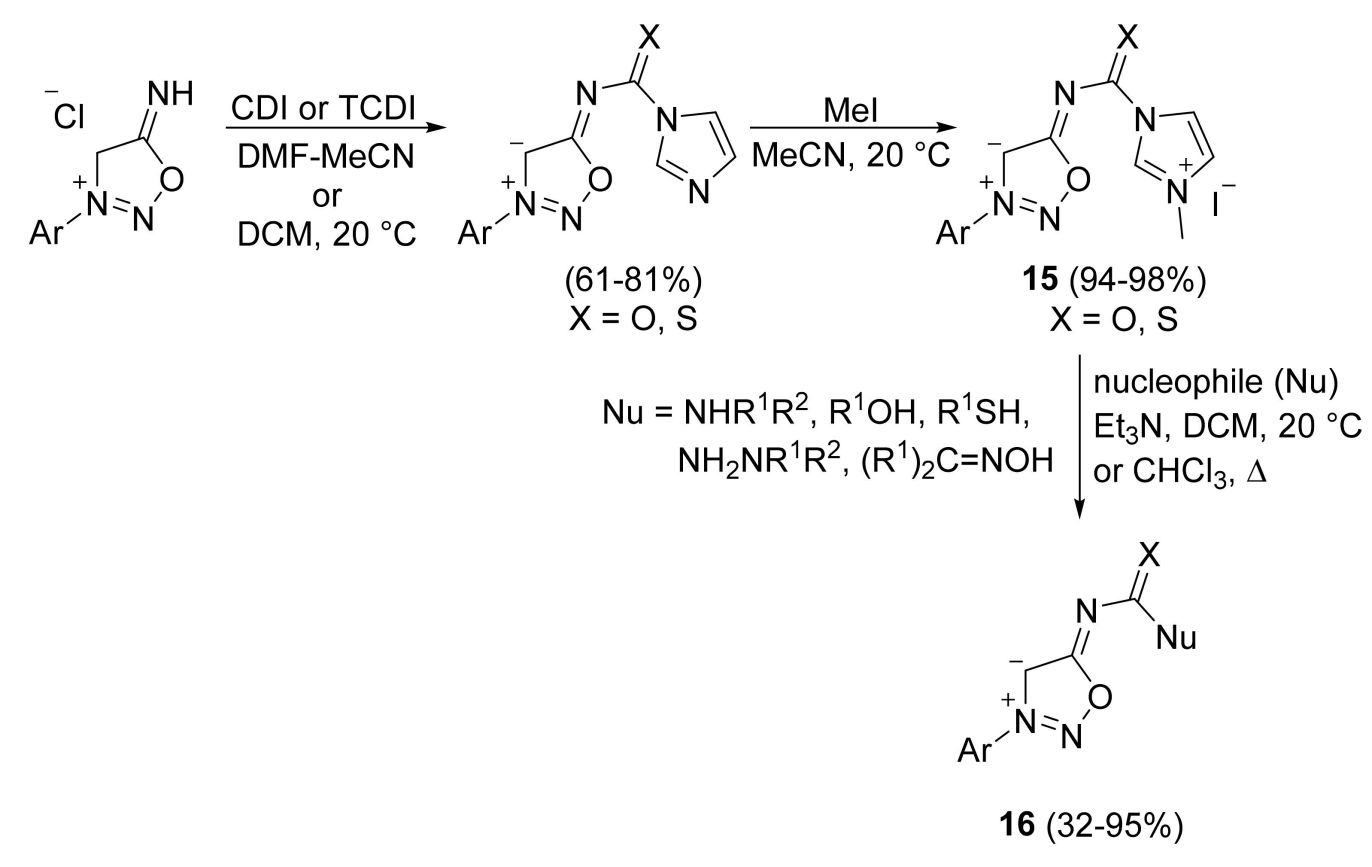
Synthesis and reactivity of carbonyl-imidazolium activated iminosydnones **15**.

A convenient methodology for the selective N^6^-exocyclic functionalization of sydnone imines involving the addition of a large variety of nucleophiles on carbonyl-imidazolium-activated iminosydnones **15** was developed [40]. Initial imidazolium derivatives **15** can be easily obtained in two steps from the corresponding sydnone imines on a multigram scale. Compounds **15** are bench-stable for several weeks at room temperature and react efficiently with diverse heteroatom nucleophiles to afford a library of functionalized sydnone imines **16** (Scheme 9) [40]. This method has a broad substrate scope and tolerates sensitive functional groups. Variety of alkyl- and aryl-substituted amines, alcohols and thiols undergo nucleophilic substitution to achieve corresponding N^6^-functionalized sydnone imines **16**.

**Scheme 10:**
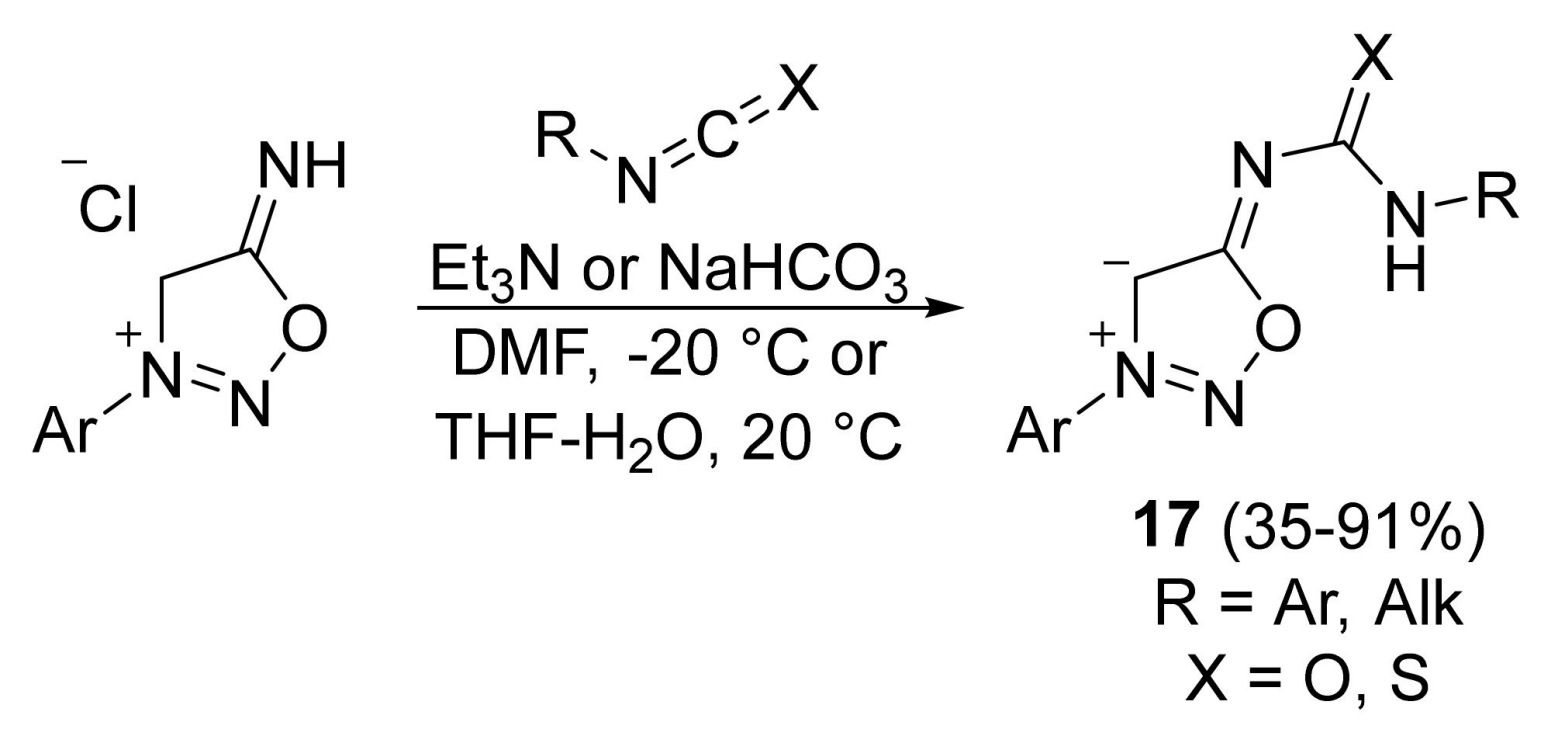
Synthesis of carbamoyl and thiocarbamoyl sydnone imines **17**.

A number of sydnone imine-based ureas and thioureas **17** were prepared by reaction of parent iminosydnones with the corresponding aromatic and aliphatic isocyanates or isothiocyanates (Scheme 10) [39].

**Scheme 11:**
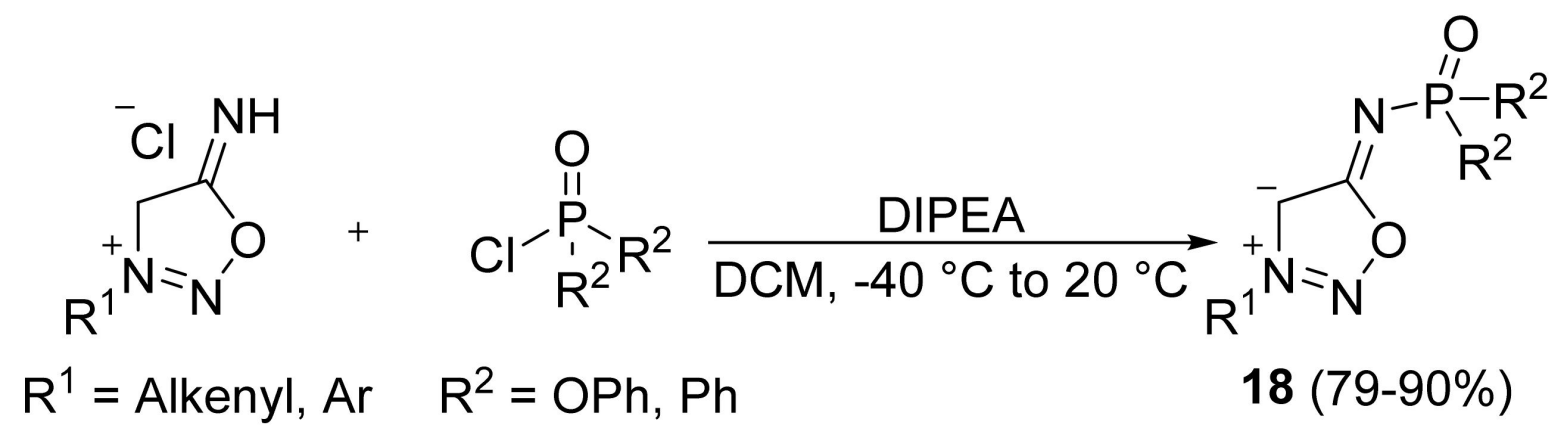
Preparation of N^6^-phosphorylated iminosydnones **18**.

An interaction of sydnone imine hydrochlorides with substituted phosphinate or phosphonate chlorides in the presence of diisopropylethylamine (DIPEA) was shown to be a highly efficient method for the preparation of N^6^-phosphorylated iminosydnones **18** in good and high yields (Scheme 11) [41]. Gram-scale quantities of N^6^-phosphorous derivatives **18** can be easily synthesized enabling their wide utilization in organic synthesis. 

**Scheme 12:**
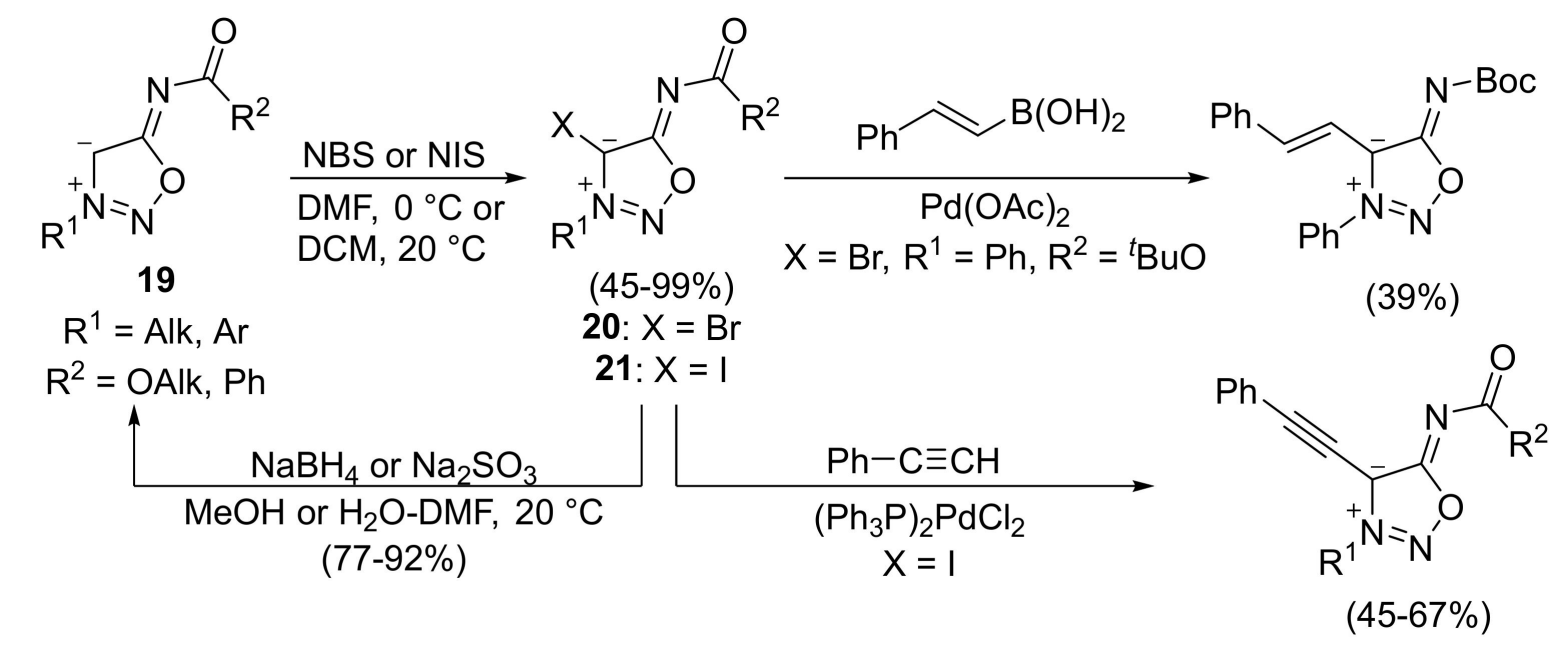
Synthesis and reactivity of halogenated sydnone imines.

C^4^-Halogenation of iminosydnones bearing a protected N^6^-imine function is one of the simplest ways for the functionalization of the sydnone imine scaffold. Sydnone imines **19** selectively halogenated at C^4^ position by treatment with *N*-bromosuccinimide (NBS) or *N*-iodosuccinimide (NIS) to form 4-bromo- or 4-iodo derivatives **20** and **21**, respectively [39,42]. Unfortunately, 4-chlorosydnone imines were not formed even in trace amounts upon utilization of *N*-chlorosuccinimide. Interestingly, debromination of bromosydnone imines **20** proceeds in high yields using NaBH_4_ or Na_2_SO_3_ [19]. The 4-bromosydnone imines **20** undergo Suzuki coupling with styrylboronic acid, while 4-iodoiminosydnones **21** react with phenylacetylene through Sonogashira coupling (Scheme 12) [39].

**Scheme 13:**
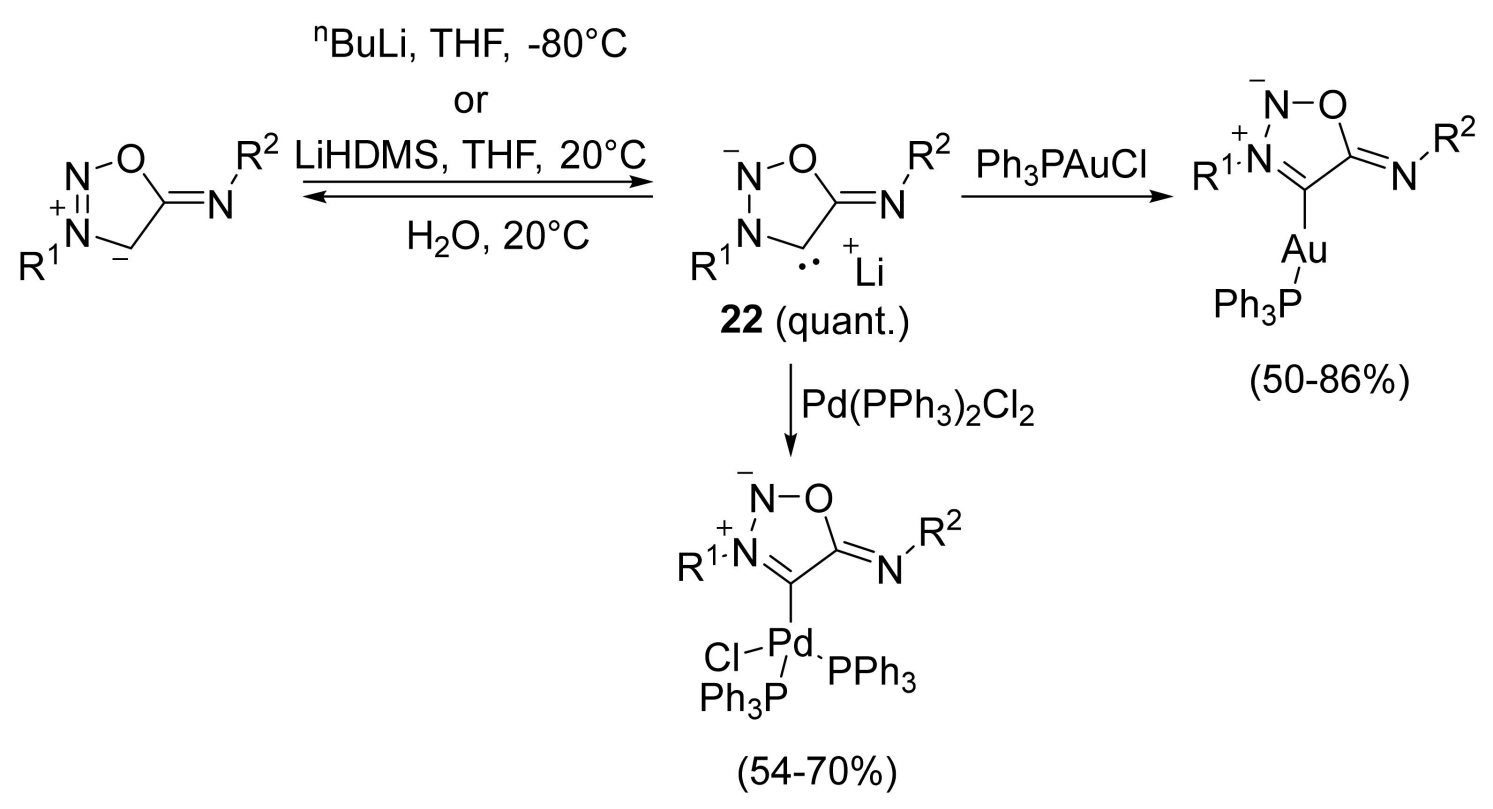
Lithiation of sydnone imines and NHC thereof.

Numerous patterns of C^4^ sydnone imine functionalization involve a preliminary lithiation of the starting material with *^n^*BuLi or LiHDMS in THF. The resulting deprotonated C^4^-lithio derivatives **22** are stable at room temperature in solutions under an inert atmosphere for several weeks and form anionic *N*-heterocyclic carbenes (NHC) (Scheme 13) capable of reaction with various electrophiles [41,43,44]. Interaction of C^4^-lithiosydnone imines with MeCN-*d*_3_ resulted in C^4^-deuterated mesoionic in quantitative yield, while the same reaction did not occur in the case of free sydnone imine. Anionic iminosydnone NHC may be trapped in a form of gold or palladium complexes by a treatment with Ph_3_PAuCl and (Ph_3_P)_2_PdCl_2_, respectively. N^3^-aryl and N^3^-morpholinylsydnone imine carbene Pd complexes were tested as catalysts in Suzuki–Miyaura and Sonogashira–Hagihara cross-coupling reactions [45].

**Scheme 14:**
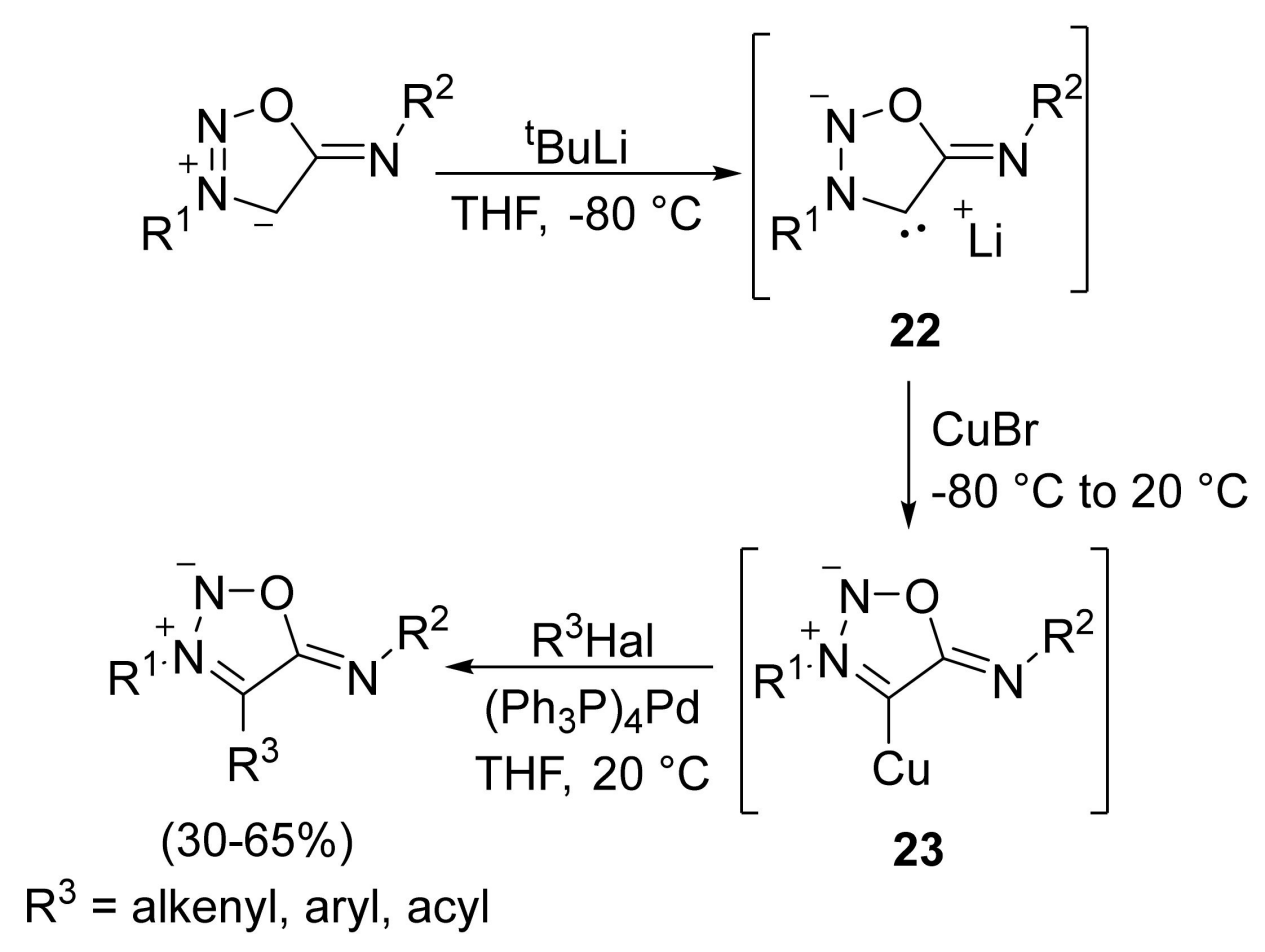
Copper-mediated cross-coupling reaction of sydnone imines with organic halides.

Sydnone imine-based copper complex as intermediate in cross-coupling reaction with various organic halides in the presence of Pd(PPh_3_)_4_ was postulated (Scheme 14) [45]. The addition of copper bromide to a solution of sydnone imine carbenoid **22** at −80 °C provides a deep-colored solution of C^4^-copper derivative **23**, which is fairly stable even at room temperature.

**Scheme 15:**
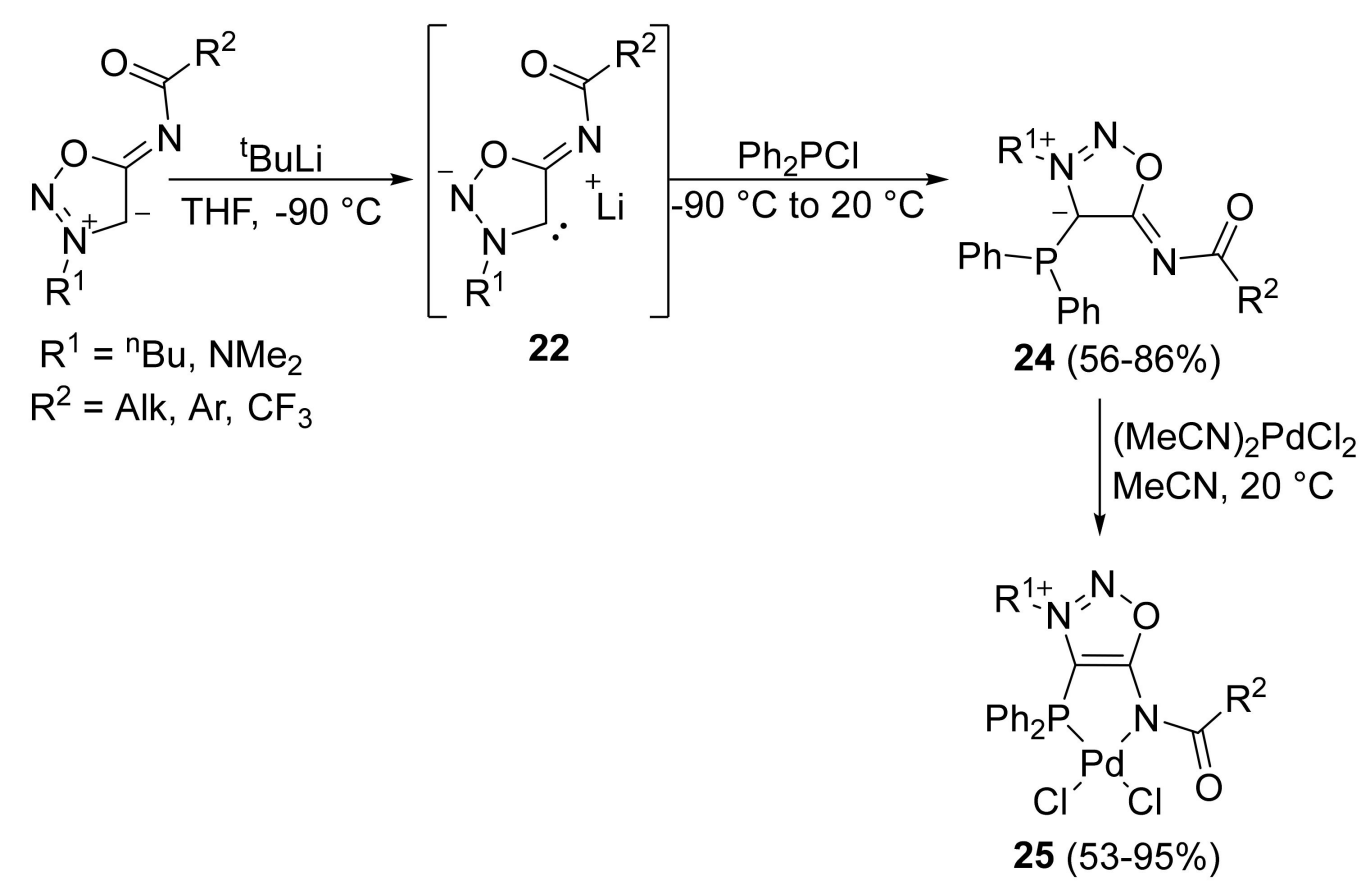
Synthesis of phosphorus-functionalized sydnone imines.

C^4^-lithiated iminosydnones **22** react with diphenylphosphine chloride leading to a formation of C^4^-phosphine substituted derivatives **24** bearing two diverse donor atoms [46]. Considering the resulting functionalized sydnone imines as hemilabile bidentate ligands they were converted to 5-membered palladium complex **25** through the reaction with PdCl_2_(MeCN)_2_ (Scheme 15). DFT calculations of charge distribution and X-ray diffraction analysis for this unusual palladium mesoionic ligand coordination were carried out [46].

**Scheme 16:**
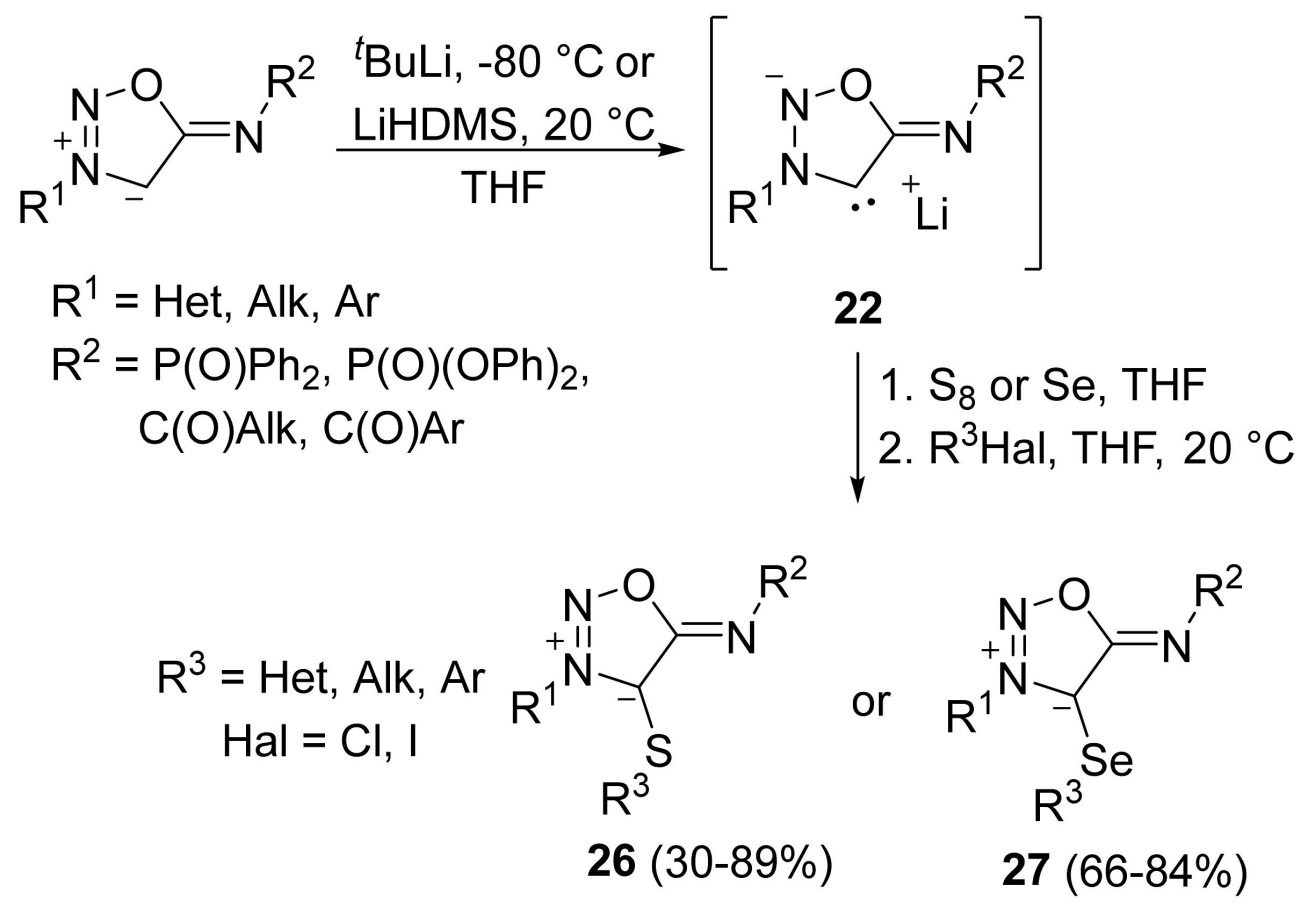
Preparation of thioether and selenoether sydnone imines.

C^4^-Thioether and C^4^-selenoether derivatives **26** and **27**, respectively, were prepared [45,47] via C^4^ carbon lithiation, interaction of carbenoids **22** with elemental sulfur or selenium followed by reaction with electrophiles such as alkyl and aryl halides (Scheme 16). Lithium thiolate intermediates proved to be unstable and decomposed quickly upon isolation or storage in solutions.

**Scheme 17:**
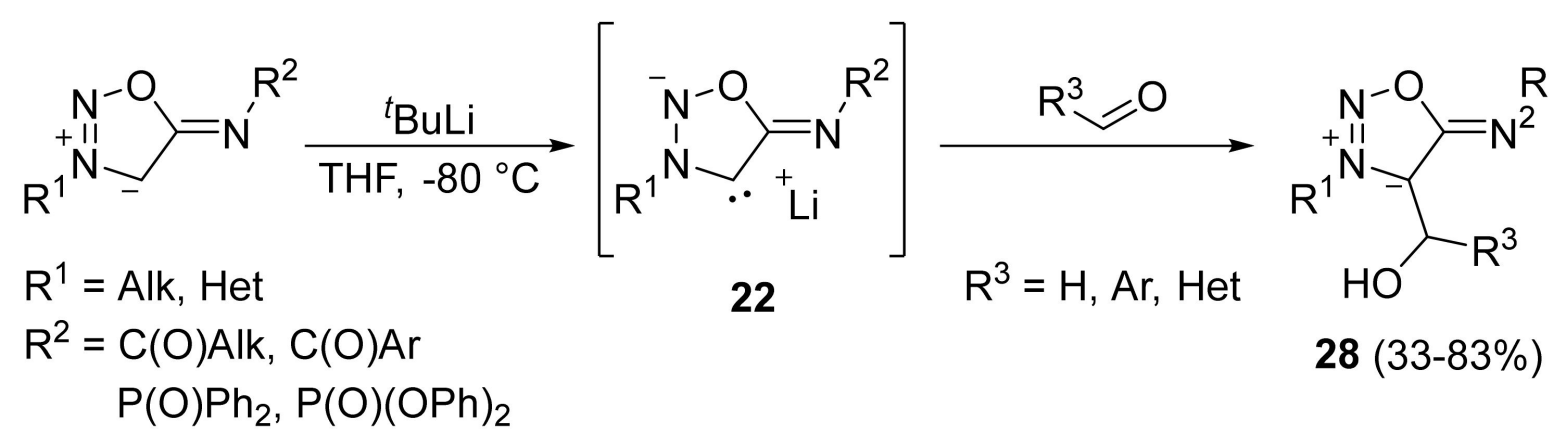
Interaction of sydnone imine carbenoids with aldehydes.

C^4^-Lithiated iminosydnones **22** add to non-enolizable carbonyl compounds to form corresponding secondary alcohols **28** (Scheme 17). Initial lithioiminosydnone carbenoids exhibit low nucleophilicity and are thermally unstable. At −80 °C, these substances did not react with active electrophiles such as trimethylsilyl chloride, methyl iodide and allyl bromide, while at higher temperatures they underwent rapid decomposition [48].

**Scheme 18:**
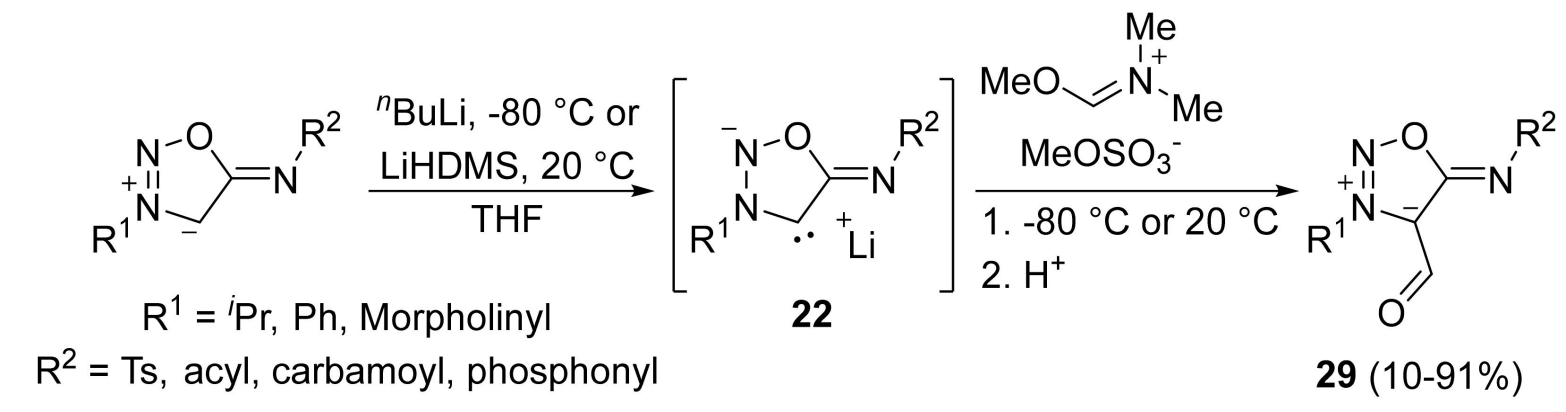
Formylation of sydnone imines.

Reaction conditions for the formulation of sydnone imines were studied using different lithium bases followed by reaction with 1-methoxy-*N*,*N*-dimethylmethyleneiminium methyl sulfate (Scheme 18) [48]. Metalation with LDA, LiHDMS and *^n^*BuLi provides higher yields of aldehydes **29** than Et_2_NLi. At the same time, reactions of C^4^-lithiated sydnone imines with DMF as well as direct formulation using the Vielsmeier reagent were unsuccessful.

**Scheme 19:**
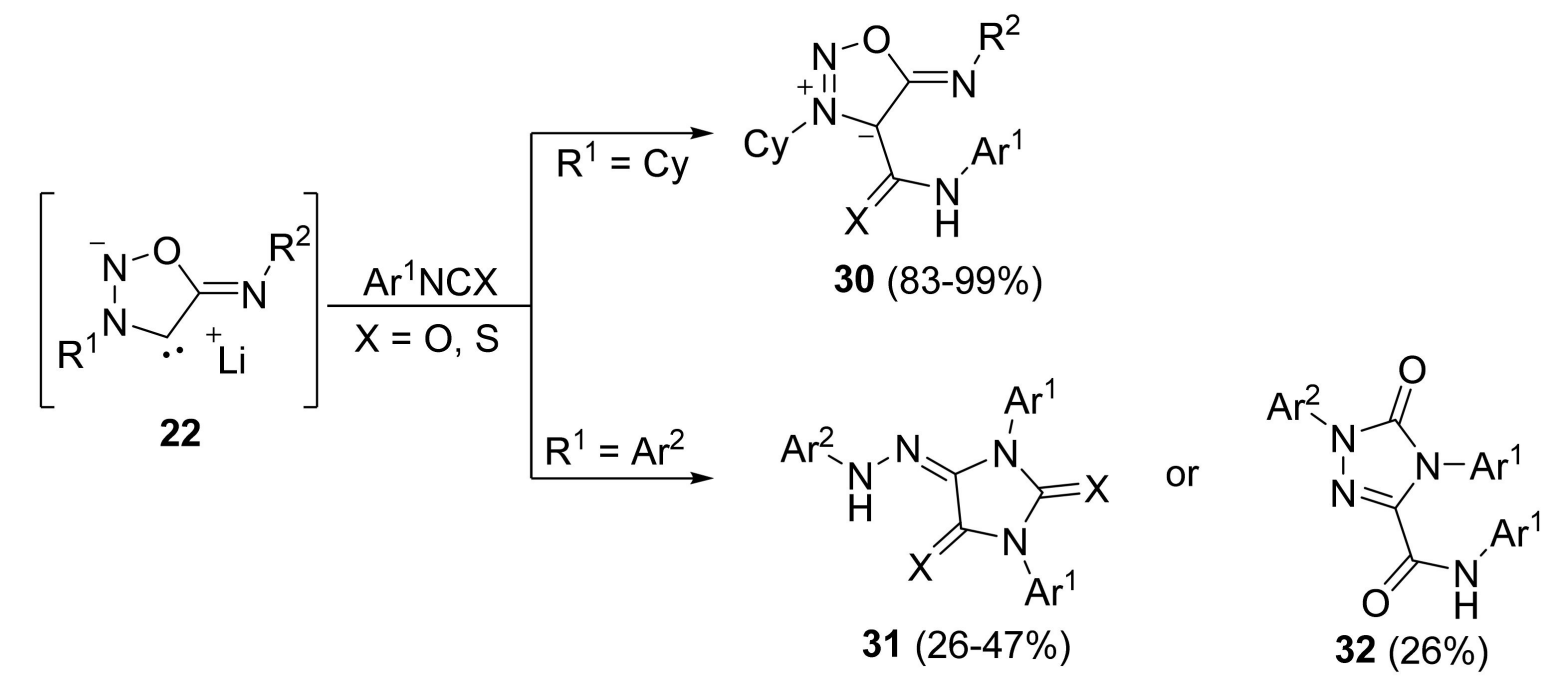
Interaction of sydnone imine carbenoids **22** with isocyanates and isothiocyanates.

Recently, it was shown that N^3^-cyclohexyl-C^4^-lithioiminosydnone can react with aryl isocyanates and isothiocyanates with a formation of the corresponding amides and thioamides **30** in high yields. However, the same reaction with N^3^-aryl-substituted sydnone imines **22** afforded 5-arylhydrazono-imidazolidine-2,4-diones or -dithiones **31** and 1,2,4-triazoles **32** in moderate yields (Scheme 19). Reaction conditions, stoichiometric amounts of isocyanates and isothiocyanates and substituent at the N^3^ position of the sydnone imine ring were shown to strongly affect the reaction pathway. Generally, utilization of isocyanates instead of isothiocyanates and conducting the reaction at room temperature promoted the formation of 1,2,4-triazoles **32** [49]. Interaction of lithio sydnone imines **22** with tetracyanoethylene afforded pyrazoles as a result of reductive 1,3-dipolar cycloaddition, while the addition of azodicarboxylate gave 4-hydrazinylsydnone imines [50]. A formation of C^4^-B and C^4^-Hg adducts from carbenoids **22** was also reported [51].

**Scheme 20:**
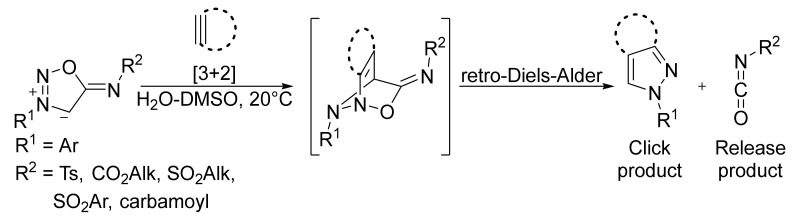
Bioorthogonal reactions of sydnone imines.

Cycloaddition reactions for mesoionic compounds were studied extensively on sydnones and münchnones, while for sydnone imines this process was investigated rarely. A [3+2]-cycloaddition of N^6^-derived iminosydnones with several strained alkynes resulted in the formation of pyrazoles and isocyanates (Scheme 20). This method is based on a general concept of bioorthogonal click-and-release reactions [36,38,39,40,52,53] due to the ability of sydnone imines to remove the mesoionic fragment through cycloaddition with alkyne triggers. It was shown that electron-withdrawing substituents at N^3^-aryl iminosydnones and a urea moiety connected to the exocyclic nitrogen atom of the iminosydnone core had a strong beneficial impact on the reaction kinetics. Moreover, sydnone imines bearing azide function have diverse reactivity towards alkynes depending on a structure of dipolarophile, which allows for performing sequential [3+2]-cycloadditions. Biorthogonal reactions of sulfonyl sydnone imines as prodrugs and dibenzoazacyclooctyne derivatives are served as an efficient tool to release sulfonamide medications under physiological conditions. An extensive and excellent review on the synthetic and biomedical applications of bioorthogonal reactions of sydnone imines and related mesoionic compounds was very recently published [54].

**Scheme 21:**
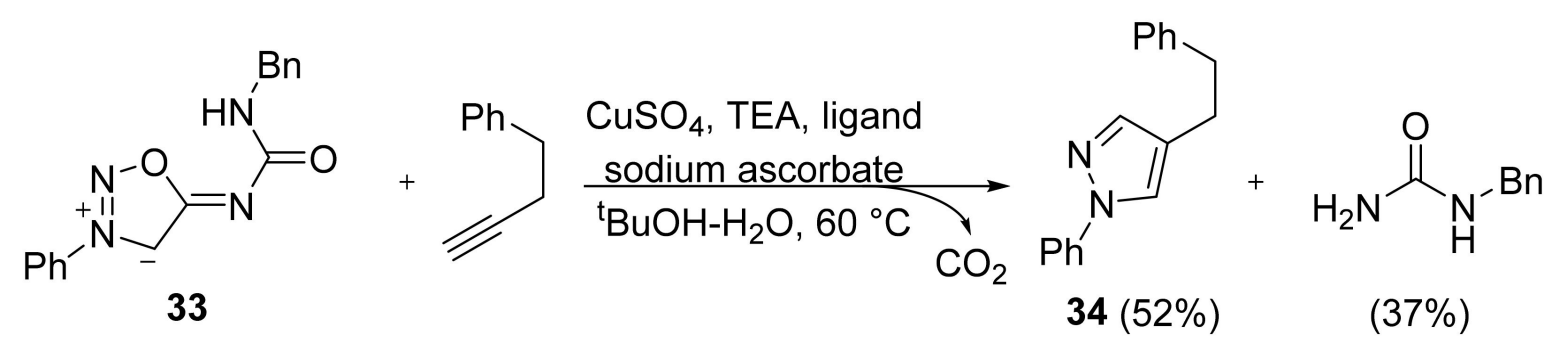
Click reaction of sydnone imine **33** with phenylbutyne.

A recent example of iminosydnone [3+2]-cycloaddition with unstrained alkyne was revealed in a copper-catalyzed reaction of terminal phenylbutyne and N^3^-phenyl-N^6^-carbamoyl sydnone imine **33** (Scheme 21) [55]. However, heating at 60 °C provided only a moderate yield of cycloaddition-retro-Diels-Alder product **34**, attempts to improve the reaction efficiency were unsuccessful.

**Figure 3:**
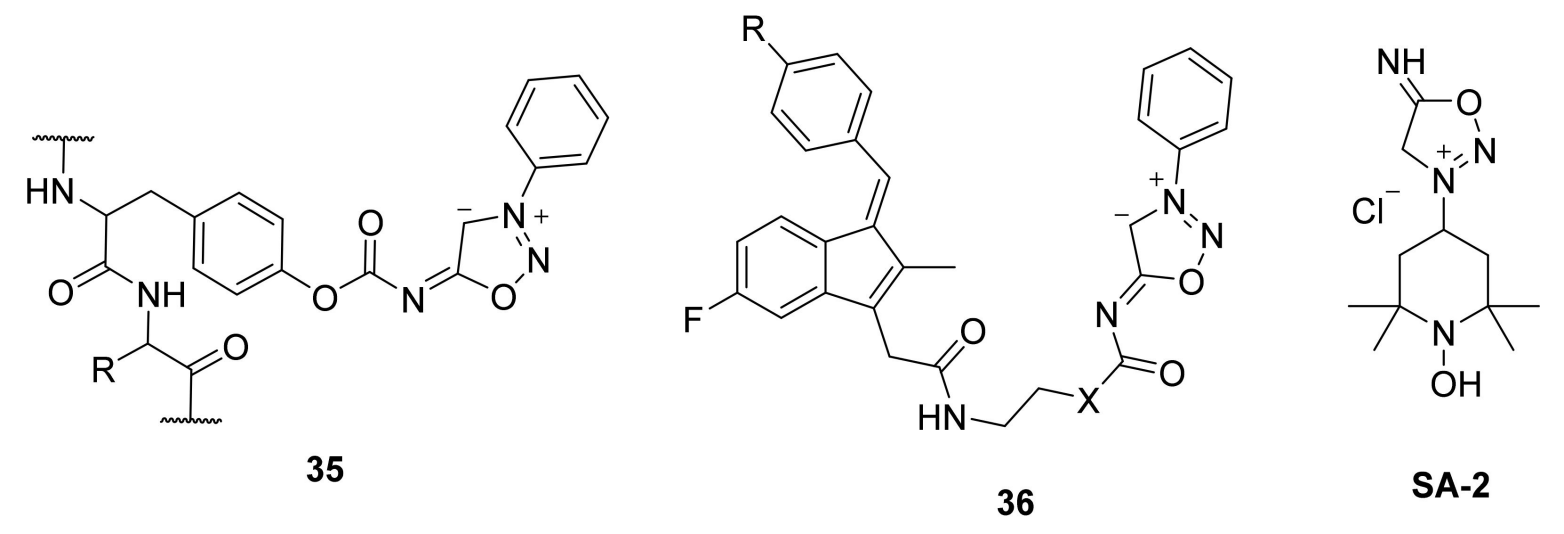
Structures of sydnone imine-based hybrids.

The most studied application of iminosydnones is their capability of exogenous NO release under physiological conditions [56]. Several sydnone imines possessing good NO-donor profiles were discovered as possible drug candidates due to their vasodilating and antihypertensive action. In vivo experiments on various mammals (dogs, cats, rabbits and pigs) showed that *N*-ethoxycarbonyl-3-morpholinosydnone imine produced a gradually developing and prolonged hypotensive action which was characterized by a decrease in pulse pressure, because of a greater fall in systolic than diastolic pressure. In addition, *N*-ethoxycarbonyl-3-morpholinosydnone imine was found to be non-toxic to animals in concentrations up to 100 μg/mL [57]. Moreover, several clinically approved iminosydnones, such as Molsidomine, revealed high antiplatelet activity. The platelet-inhibiting effect is presumably associated with direct activation of platelet-soluble guanylate cyclase by **SIN-1**, the bioactive metabolite of molsidomine [58]. Design and synthesis of a series of sydnone imine amino acids conjugate **35** with regard to the development of peptide-based therapies in medicinal chemistry were also carried out. Prepared sydnone imine amino acids hybrids showed moderate levels of NO release in a glutathione (GST) buffer containing superoxide dismutase (SOD), while similar studies under identical conditions in the absence of GST and SOD gave no significant NO production [59]. Prepared amino acids were shown to be exogenous pro-NO-release compounds that allow for improving the strategy of localization and targeting of NO-delivery. Molecular hybridization of a sydnone imine scaffold with integrin binding aspartic acid-glycine-arginine (RGD) peptide sequence and one of the chemotherapeutic agents, abiraterone, resulted in increased cytotoxic effects against PC3 and MCF7 cancer cell lines [60]. An analogous approach was used for a combination of the iminosydnone motif with known NSAID sulindac which improved antiproliferative and anti-inflammatory properties of the resulted hybrids **36** (Figure 3). These sydnone imine-sulindac conjugates showed promising cytotoxic activity at a concentration of 50 μM, while at lower concentrations (1.0 and 0.5 μM) no measurable cytotoxic effects were detected [61]. Dual acting NO-donor-antioxidant sydnone imine **SA-2** bearing 1-hydroxy-2,2,6,6-tetramethylpiperidine moiety revealed an ability to protect photoreceptor cells from H_2_O_2_ induced oxidative stress and may promote an intraocular pressure lowering [34]. In addition, sydnone imines demonstrate a number of other pharmacological activities including psychostimulant [62] and antibacterial [63], as well as being found to act as trypanocidal agents [64]. The lethal dose (LD_100_) of sydnone imines in *Trypanosoma equiperdum* was determined to be 25 and 50 μM within 48 h, respectively [64]. Using a combination of molecular docking and molecular dynamics simulations a series of iminosydnone-based insecticides was designed and synthesized [65]. Aside from biomedical applications, sydnone imine derivatives were used as additives or catalysts in Pd-catalyzed cross-coupling reactions [66,67]. Additionally, recently, the first representatives of sydnone imine-based dense energetic materials with high detonation performance were prepared [68].

## 3. Furoxans

**Scheme 22:**
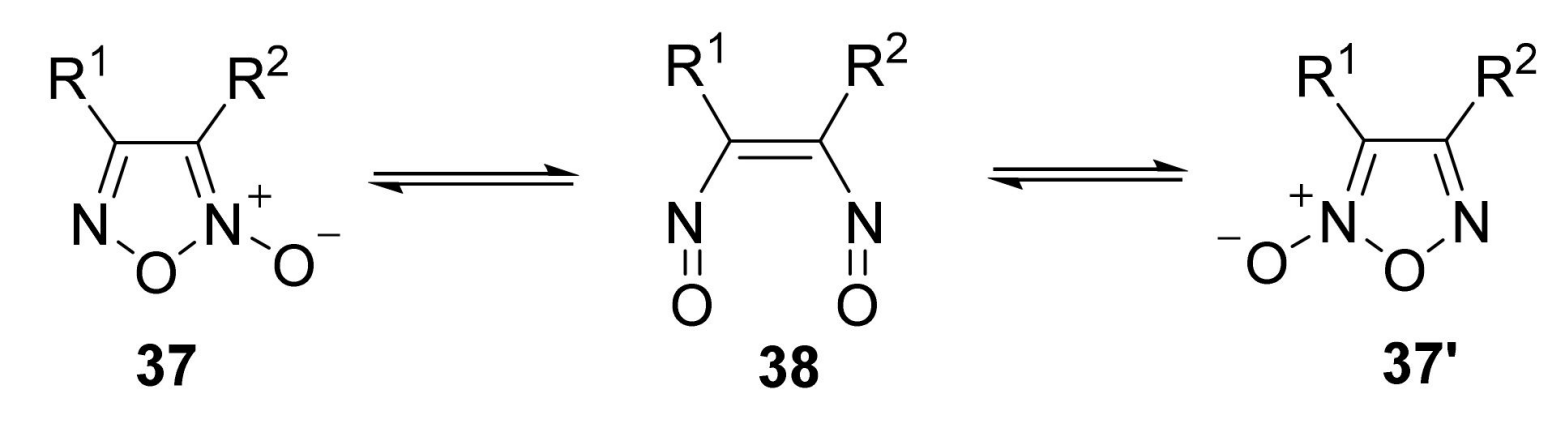
Mechanism of the furoxan ring isomerization.

Among the variety of nitrogen–oxygen organic and organometallic compounds capable of NO release under physiological conditions, the furoxan (1,2,5-oxadiazole 2-oxide) scaffold has attracted considerable attention [69,70,71,72] mainly due to the high stability of the furoxan cycle under ambient conditions and absence of nitrate tolerance under continuous therapy [23]. Long-term investigations of Prof. Alberto Gasco [73,74] revealed a possibility of regioisomeric furoxans with different positions of the *N*-oxide group to produce NO in significantly different amounts. Since furoxan isomers **37** and **37′** can be interconverted to each other through dinitrosoethylene intermediate **38** at heating or photoirradiation (Scheme 22), this important feature may lead to the diversity-oriented construction of novel NO-donor drug candidates depending on the structure of furoxan isomer and biological target.

**Figure 4:**
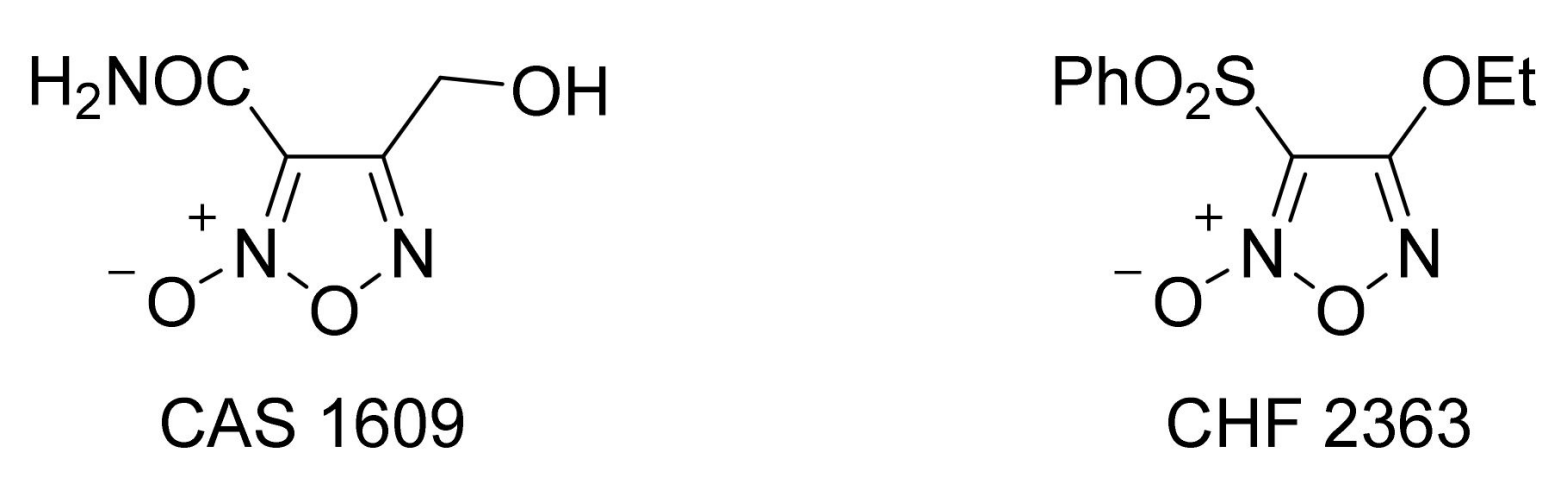
Structures of CAS 1609 and CHF 2363.

The two most promising NO-donor furoxans–CAS 1609 and CHF 2363 (Figure 4)–that possess vasodilating and anti-aggregation properties were discovered in the mid-1990s. CAS 1609 is a strong NO donor, which significantly increases cGMP levels in animal models of pulmonary artery strips and at low doses, decreases blood pressure and left ventricular end-diastolic pressure [75]. Later, our group revealed significant antiaggregant activity of CAS 1609 induced by adenosine diphosphate (ADP) and adrenaline and partially by collagen [76]. Similar cardiovascular properties were displayed by CHF 2363, which exhibits significant NO-release capacity and exerts anti-aggregation and vasodilating activity [77]. At the same time, CAS 1609 displays moderate cytotoxic and genotoxic effects at very high concentrations (1 mM), whereas similar effects of the water-soluble analog of CHF 2363 start to occur at much lower concentrations (5 μM). It was clearly shown that such effects are closely associated with the NO-donor capacity of furoxans by comparison with non-NO-donating furazans, and because both effects are decreased in the presence of the NO scavenger oxyhemoglobin [78].

**Scheme 23:**
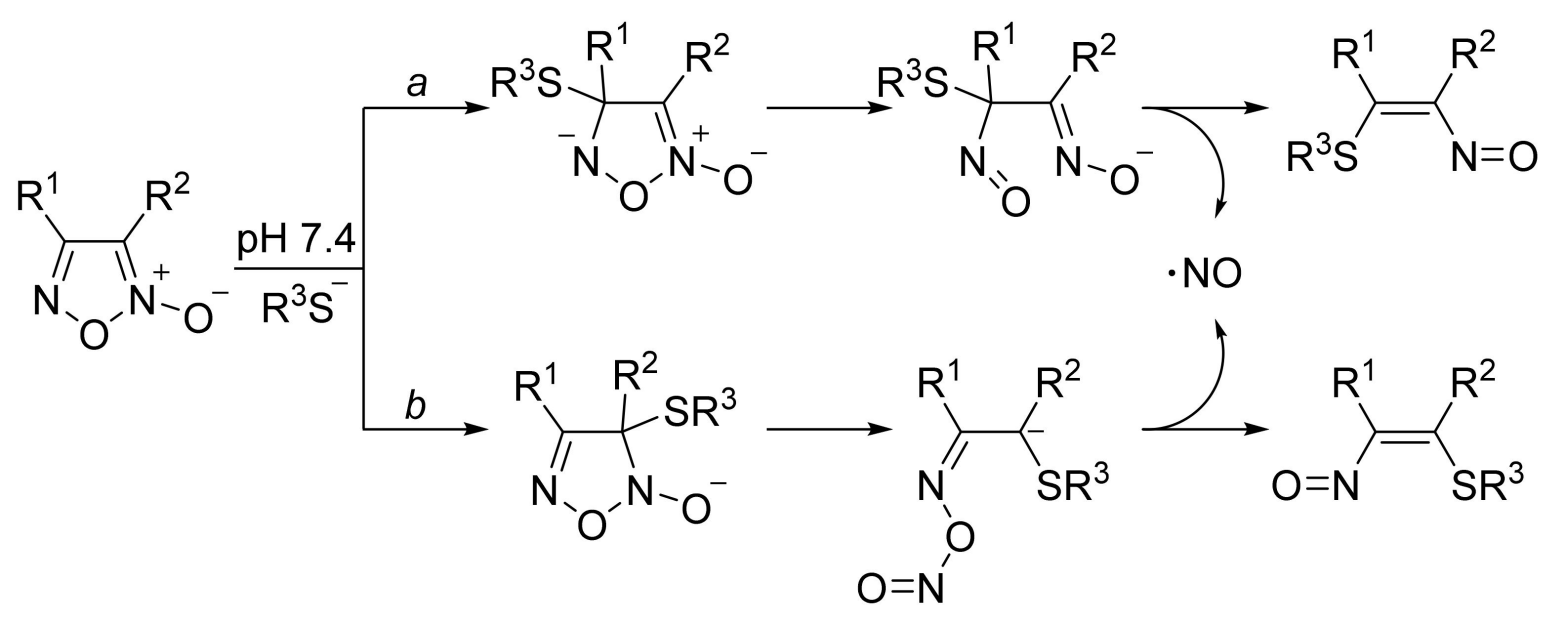
Mechanism of NO release from furoxans.

The thiol-dependent mechanism of NO-release from furoxans is now widely accepted, however, details of this process remain uncertain. Since the furoxan ring has a strong electron-withdrawing character, both carbon atoms of this heterocycle are quite electrophilic and are prone to reaction with nucleophiles. Thus, there are two main routes resulting in NO-release from furoxans depending on whether thiolate-anion, generated, for example, from cysteine, attacks C(3) or C(4) atom of the furoxan ring (Scheme 23). Both degradation pathways *a* and *b* result in a furoxan ring cleavage with a concomitant release of NO. Recent mechanistic investigations [70] indicate that at least in several particular cases attack of thiolate-anion on the C(3) atom of the furoxan cycle is more favorable. Interestingly, most of the 4-nitrofuroxans do not cleave under the action of thiolate-anions but undergo nucleophilic substitution of the nitro group resulting in stable sulfanylfuroxan derivatives [69].

In recent years, the main efforts of medicinal chemists were directed towards the construction of hybrid drug candidates containing NO-donor furoxan moiety connected to a known pharmaceutical or a potential pharmacophore through an appropriate linker. However, synthetic methodologies toward regio- and chemoselective functionalization of the furoxan ring itself were also developed. In this chapter, both these approaches to the synthesis of pharmacologically active furoxan-based drug candidates are considered. Methods for the synthesis of common functionally substituted furoxans were extensively covered in some previous reviews [69,71].

**Scheme 24:**
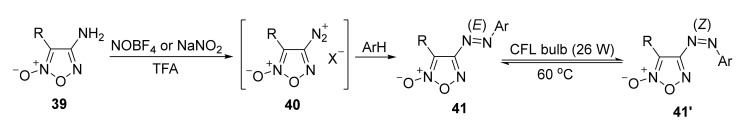
Synthesis and photoisomerization of arylazofuroxans **41**.

Recently, a new method for the selective diazotization of the readily available 4-aminofuroxans **39** [79] using NOBF_4_ as a mild nitrosating agent was developed [80]. This protocol substantially broadened the scope of furoxanyl diazonium salts **40** which can be isolated in solid-state or undergo subsequent azo coupling with electron-donating arenes or CH-acids [80]. In some cases, utilization of NaNO_2_ for diazotization was also suitable [81]. Thus formed arylazofuroxans **41** demonstrated a photoswitching ability: under visible light irradiation *E*-isomers **41** underwent an isomerization of the N=N bond generating *Z*-arylazofuroxans **41′** (Scheme 24) which are stable under ambient conditions [81]. As for most molecular photoswitches, such isomerization is equilibrium and in this case, the *E*/*Z* ratios strongly depend on both substituents at the furoxan ring and N=N bond. At heating *Z*-isomers **41′** smoothly revert to the initial *E*-arylazofuroxans **41**. Importantly, *E*-isomers **41** released low amounts of NO (<10%), while mixtures of *E*- and *Z*-isomers provided significantly higher levels of NO release (33–52%) exceeding that of reference CAS 1609. This feature is rather advantageous, especially since synthesized furoxan-based molecular photoswitches undergo photoisomerization under visible light irradiation to avoid any hazards caused by UV light.

**Scheme 25:**
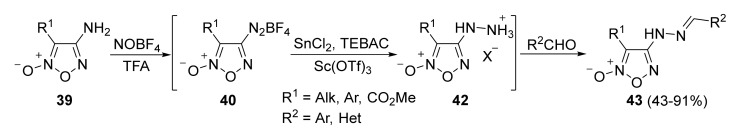
Synthesis of *N*-(furoxanyl)hydrazones **43**.

Furoxanyl diazonium salts **40** also underwent in situ reduction to the corresponding hydrazines **42** which could not be isolated due to the highly electrophilic nature of the ring but were trapped with various aldehydes to form previously unknown *N*-(furoxanyl)hydrazones **43** (Scheme 25) [82]. A utilization of TEBAC as a phase transfer catalyst and Sc(OTf)_3_ as a Lewis acid were crucial to increase the rate of the reduction and to avoid degradation of highly reactive diazonium salts **40**. A synthesized library of furoxan-based compounds represents an isosteric replacement of described drug candidates for the treatment of various neglected diseases including tuberculosis, leishmaniosis, schistosomiasis, and Chagas’ disease [83,84,85,86].

**Scheme 26:**
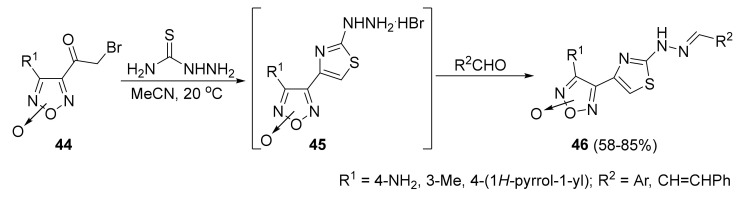
Synthesis of hydrazones based on a (2-hydrazinylthiazol-4-yl)furoxan core.

A series of hetarylfuroxans was synthesized through cyclocondensation of bromoacetylfuroxans **44** with various binucleophiles [87,88,89]. In particular, a regioselective condensation of substrates **44** with thiosemicarbazide afforded hydrazinylthiazoles **45** which underwent in situ reaction with aldehydes resulting in a formation of the corresponding hydrazones **46** (Scheme 26). Compounds **46** showed moderate cytotoxic activity against two human cancer cell lines A549 and HCT116 [90].

**Scheme 27:**
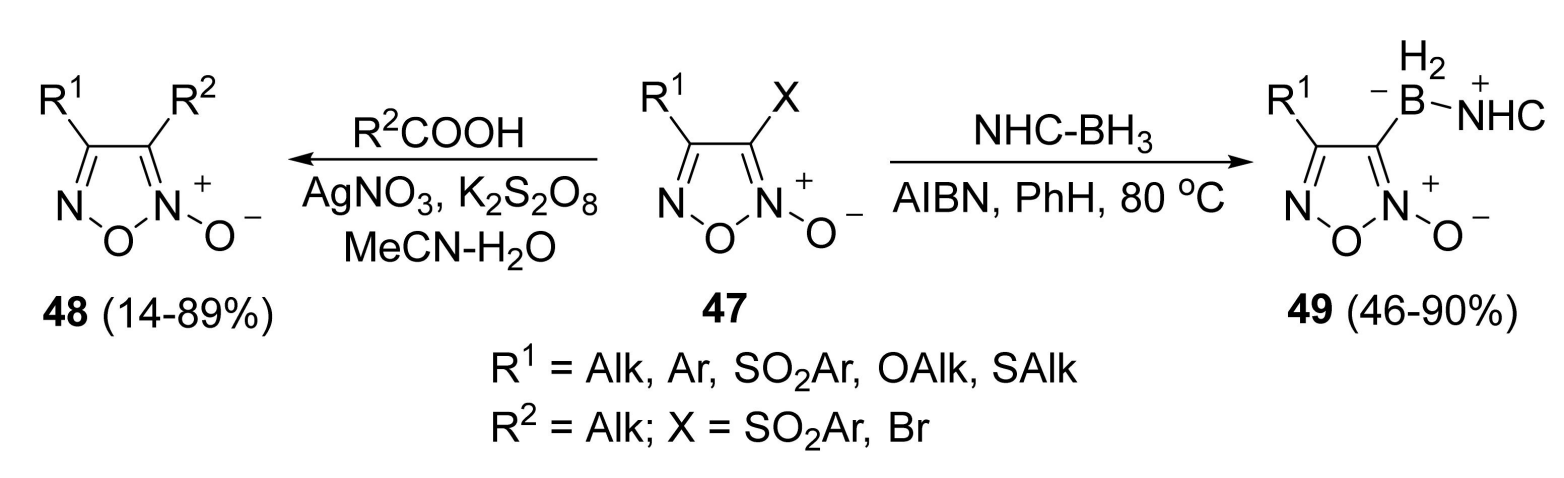
Radical functionalization of furoxans.

Recently, a novel reactivity pattern of furoxans based on their radical functionalization was established (Scheme 27). It was found that alkyl radicals generated from carboxylic acids in mild conditions add to the C(3) carbon atom of the furoxan ring in corresponding bromo or arylsulfonyl derivatives **47** followed by elimination of functional moiety and formation of target products **48** [91,92]. This protocol enables wide opportunities for the design of pharmacologically oriented furoxan-based compounds. However, it requires utilization of aliphatic carboxylic acids, while in the case of benzoic or heteroaromatic carboxylic acids target furoxans are formed in very low yields. At the same time, such methodology was also useful for the C-B bond formation directly on the furoxan ring for the synthesis of previously unknown borylfuroxans **49**. Using this approach, various *N*-heterocyclic carbene (NHC) moieties (imidazole, benzimidazole, 1,2,4-triazole and pyridine) were successfully installed via BH_2_ linkage [93].

**Scheme 28:**
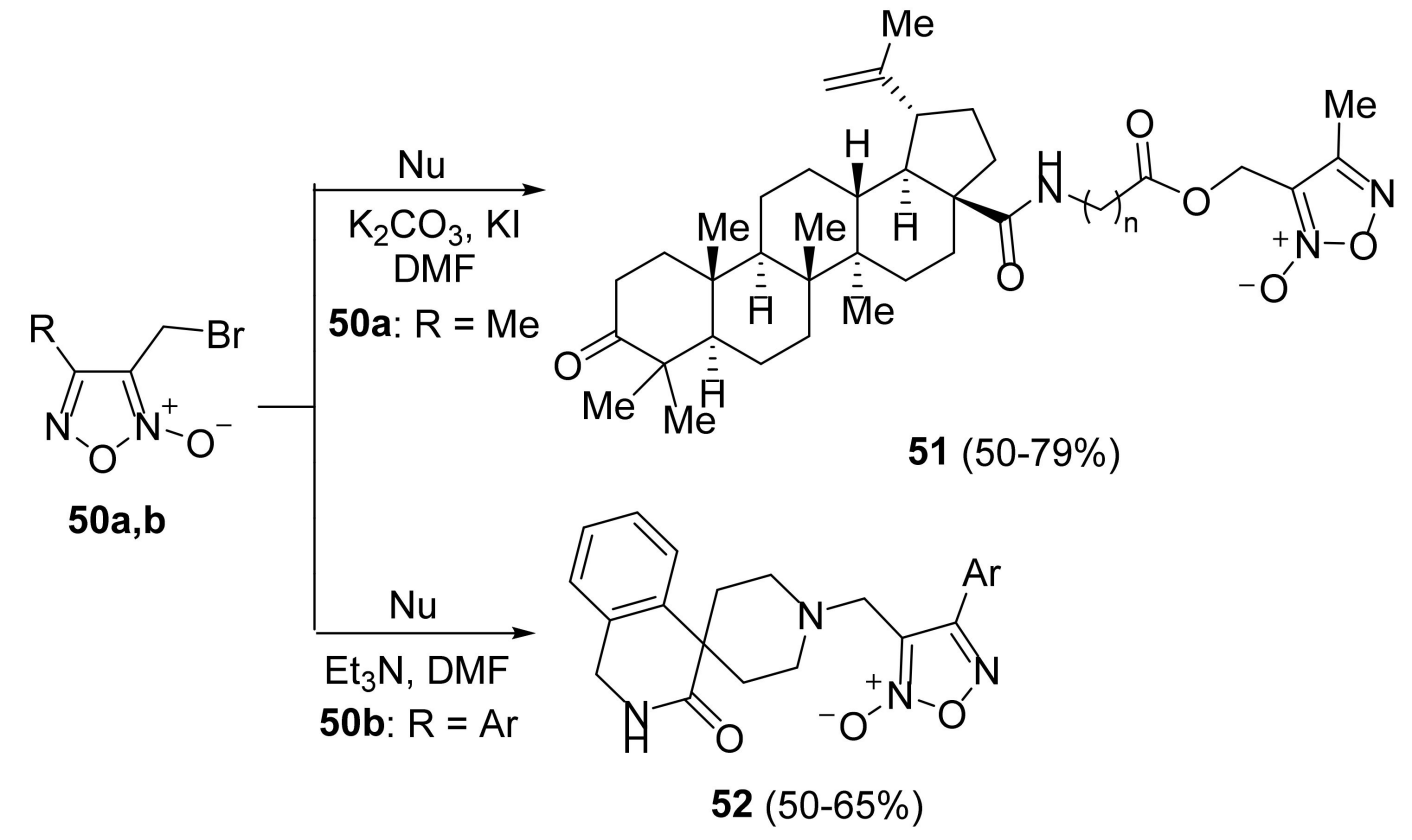
Synthesis of furoxan-based hybrids from 3-bromomethylfuroxans.

A number of hybrid drug candidates comprising of a known pharmacologically active scaffold linked to the furoxan ring directly or via an appropriate spacer were synthesized from bromomethyl- or (phenylsulfonyl)furoxans using a nucleophilic substitution reaction. For example, an interaction of 3-bromomethyl-4-methylfuroxan **50a** with amino acid-functionalized betulonic acid derivatives afforded hybrid compounds **51**, which showed moderate anti-inflammatory activity. Advantageously, most of the synthesized hybrids were non-toxic (GI_50_ > 100 μM) according to the MTT test against immortalized human fibroblasts, while reference drug doxorubicin possessed high toxicity (GI_50_ > 3.15 μM) under the same conditions [94]. The analogous reaction of 4-aryl-3-bromomethylfuroxans **50b** with spiro[isoquinoline-4,4’]-piperidine derivative resulted in a formation of the tricyclic compounds **52** (Scheme 28). All synthesized hybrids **52** demonstrated moderate to good vasodilating and antioxidant properties, while structures containing *o*-nitro- or *o*-methoxyphenyl substituents at the furoxan ring were moderate phosphodiesterase 5 (PDE 5) inhibitors as compared to the known pharmaceutical Sildenafil [95].

**Scheme 29:**
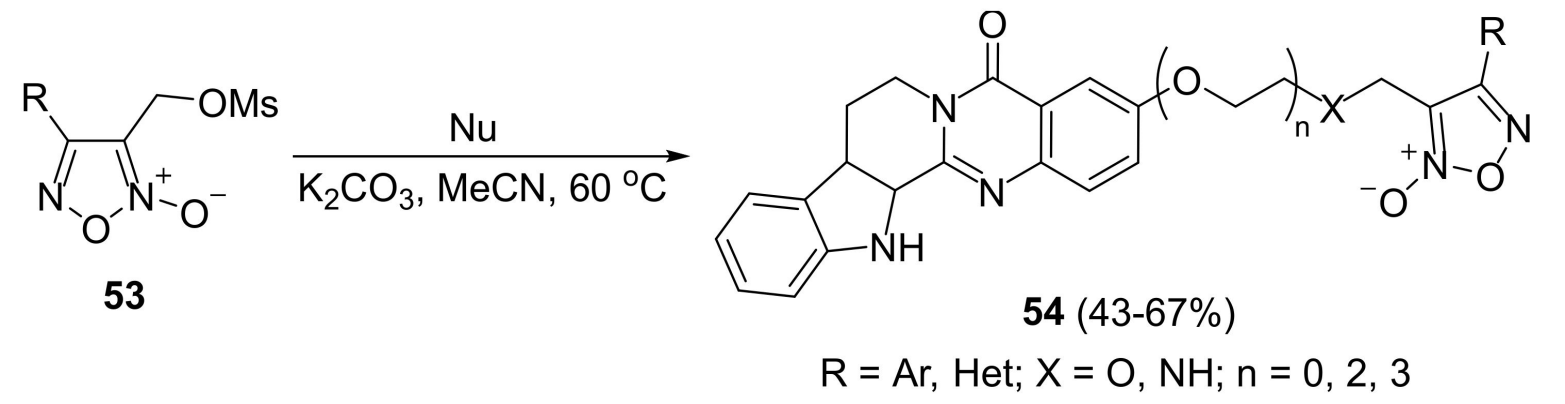
Synthesis of furoxan–rutaecarpine hybrids.

Nucleophilic substitution of the mesyloxy moiety in furoxans **53** with rutaecarpine derivatives afforded a series of hybrid structures **54** (Scheme 29). Rutaecarpine is a quinazolinocarboline-type alkaloid which is a major bioactive constituent in *Evodiae Fructus* prescribed for treatment of hypertension in traditional Chinese medicine. Molecular hybridization of this alkaloid with a furoxan subunit resulted in a more potent vasodilator and antihypertensive effect of the prepared hybrids at various dosages (20–40 mg/kg) in vivo in rats compared to parent medication [96].

**Scheme 30:**
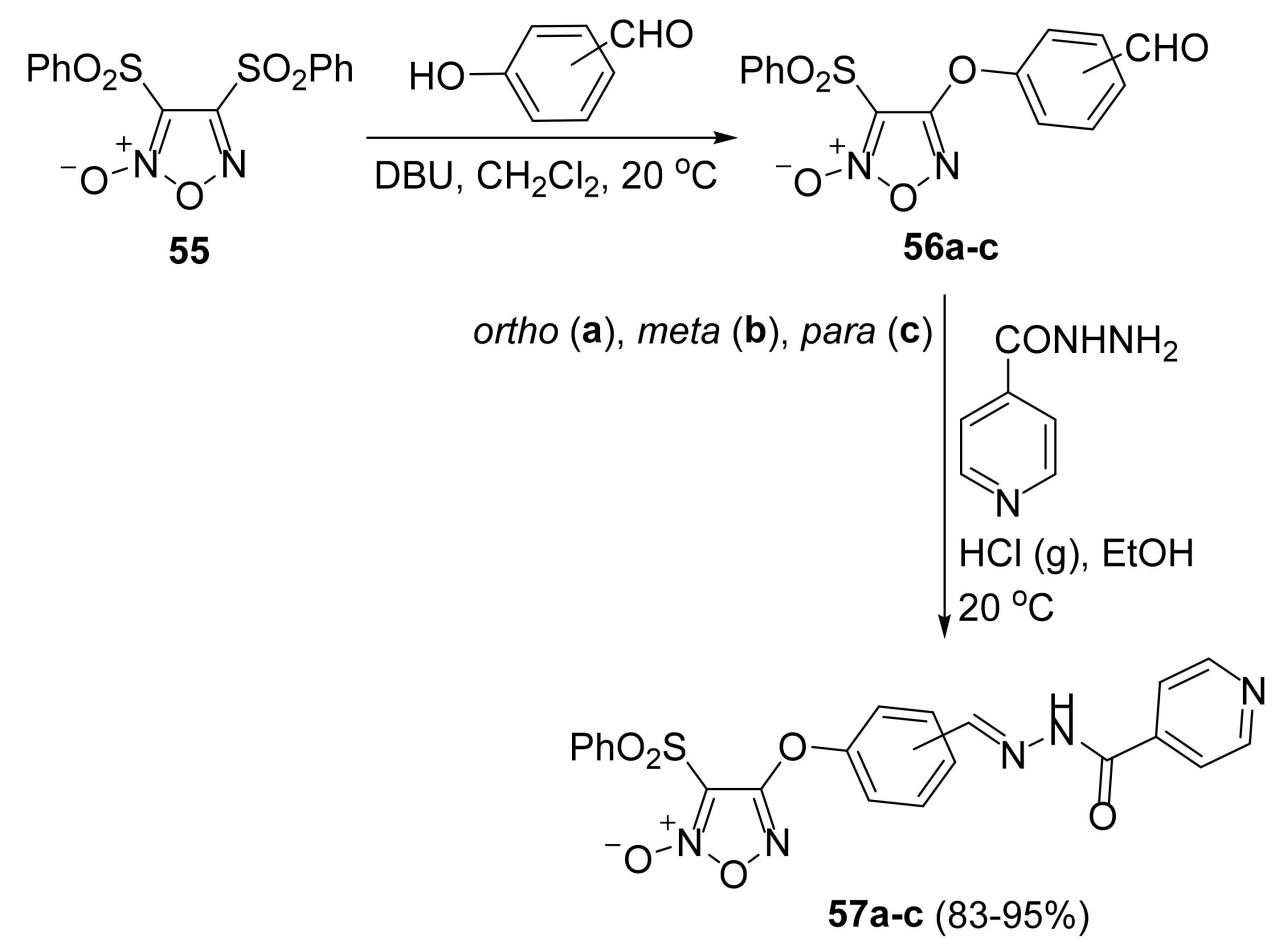
Synthesis of furoxan–hydrazone hybrids **57** linked via phenol bridge.

Due to the high electron-withdrawing nature of the furoxan ring, the S_N_Ar process is a quite versatile reaction in furoxan chemistry. As a rule, bis(phenylsulfonyl)furoxan **55** is used as a starting material for numerous transformations. Since C(4) carbon atom of the furoxan ring is much more electrophilic than C(3) atom, 4-PhSO_2_ moiety in compound **55** displaced completely selectively in reactions with nucleophiles. For example, the tandem nucleophilic substitution of a phenylsulfonyl group in furoxan **55** under the action of phenolic aldehydes with subsequent condensation of formyl group in compounds **56a–c** with isonicotinic hydrazide afforded a series of hybrids **57a–c**, which are considered as promising agents active against *Mycobacterium tuberculosis* (Scheme 30). Moreover, there is a direct correlation between NO-donor capability and antitubercular activity: the more powerful NO-donor hybrid among the series is the most active antitubercular agent [97]. Further in-depth studies revealed that compounds **57b,c** showed no mutagenicity and were active against both mono- and multidrug-resistant tuberculosis strains. In addition, treatment with furoxans **57b,c** led to undetectable levels of the bacterium in the lungs of mice while no standard drugs have shown this advantageous effect. Oral administration of furoxans to mice at 20 mg/kg dosage had no effect on behavioral parameters (Hippocratic screening) and did not damage the liver and kidneys according to the histopathology analysis [98].

A combination of NO-donor furoxan scaffold with anticancer drugs may serve as a promising tool to overcome the problem of multidrug resistance. A tumor-specific NO-release system comprising of 10-hydroxycamptothecin (HCPT)-loaded charge-reversal chitosan nanoparticles and covalently linked phenylsulfonylfuroxan on the surface was recently designed. In vitro studies proved that such a system significantly enhanced cellular uptake at pH 6.5 and immensely reduced the expression of P-glycoprotein, which was in favor of increasing killing ability against MCF-7/ADR cancer cells. Furthermore, in vivo studies confirmed that the resultant system precisely released NO in the tumor and scarcely released NO during blood circulation, avoiding NO poisoning [99].

**Scheme 31:**
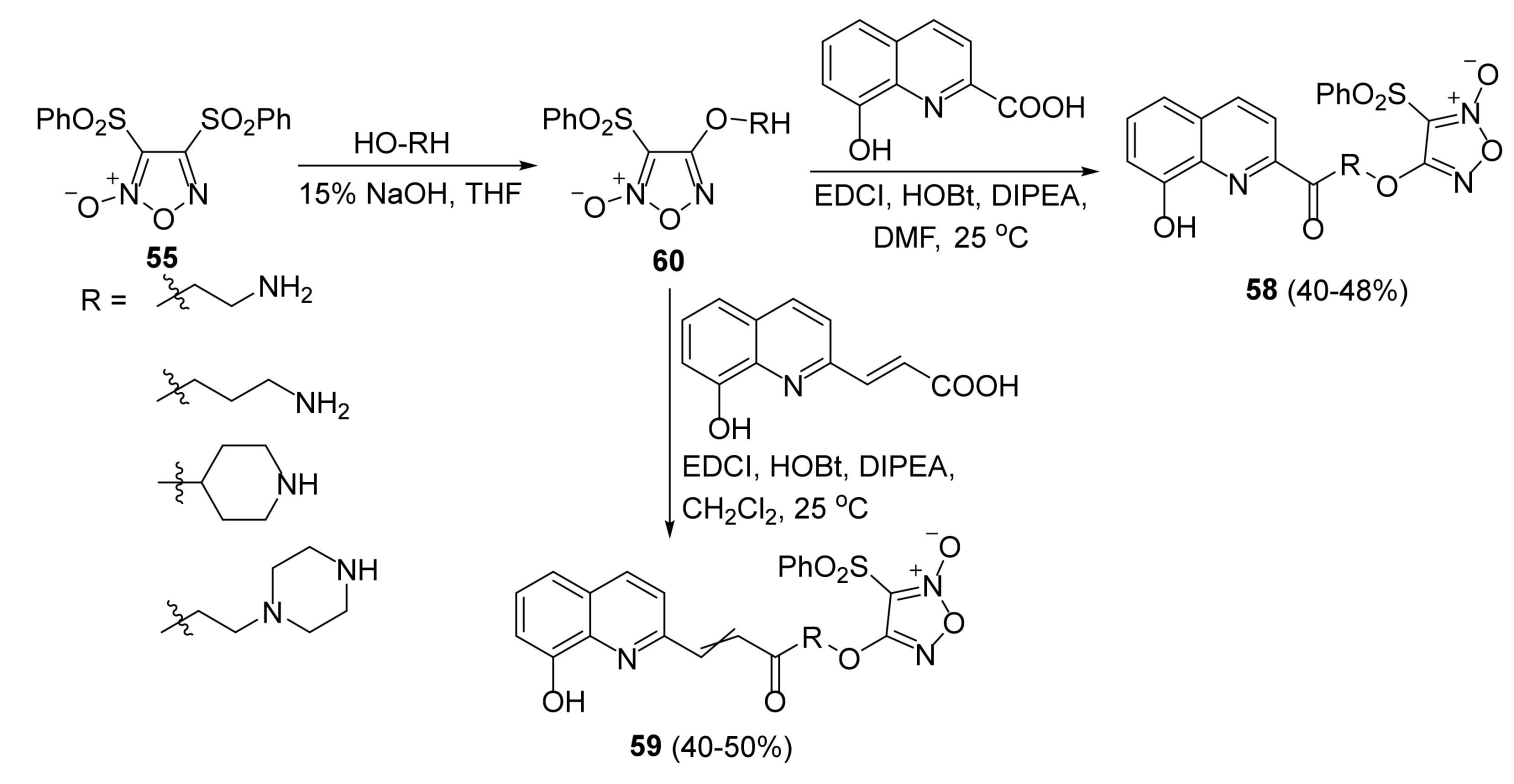
Synthesis of furoxan–hydroxyquinoline prochelator hybrids.

Due to a known chelating effect of the 8-hydroxyquinoline scaffold and its potential in cancer treatment, a series of furoxan–hydroxyquinoline hybrids **58** and **59** were synthesized through a two-step procedure. Bis(phenylsulfonyl)furoxan **55** was converted into amines **60** which underwent coupling with 8-hydroxyquinoline-based carboxylic acids (Scheme 31). Thus obtained conjugates possessed good antiproliferative activity against various cancer lines and showed good metal chelation and NO-releasing abilities. Synthesized hybrid structures demonstrated a synergistic effect caused by the presence of the furoxan motif: at the same dose of 20 mg/kg in mice, the tumor inhibition rate of a lead hybrid was 61.8%compared to 44.7% for the parent 8-hydroxyquinoline [100]. A number of various hybrid drug candidates comprising of a furoxan motif and other pharmaceutical scaffolds (estradiol [101], rhodamine B [102], triptolide [103]) were also prepared starting from bis(phenylsulfonyl)furoxan and these compounds showed promising anticancer and anti-inflammatory properties.

**Figure 5:**
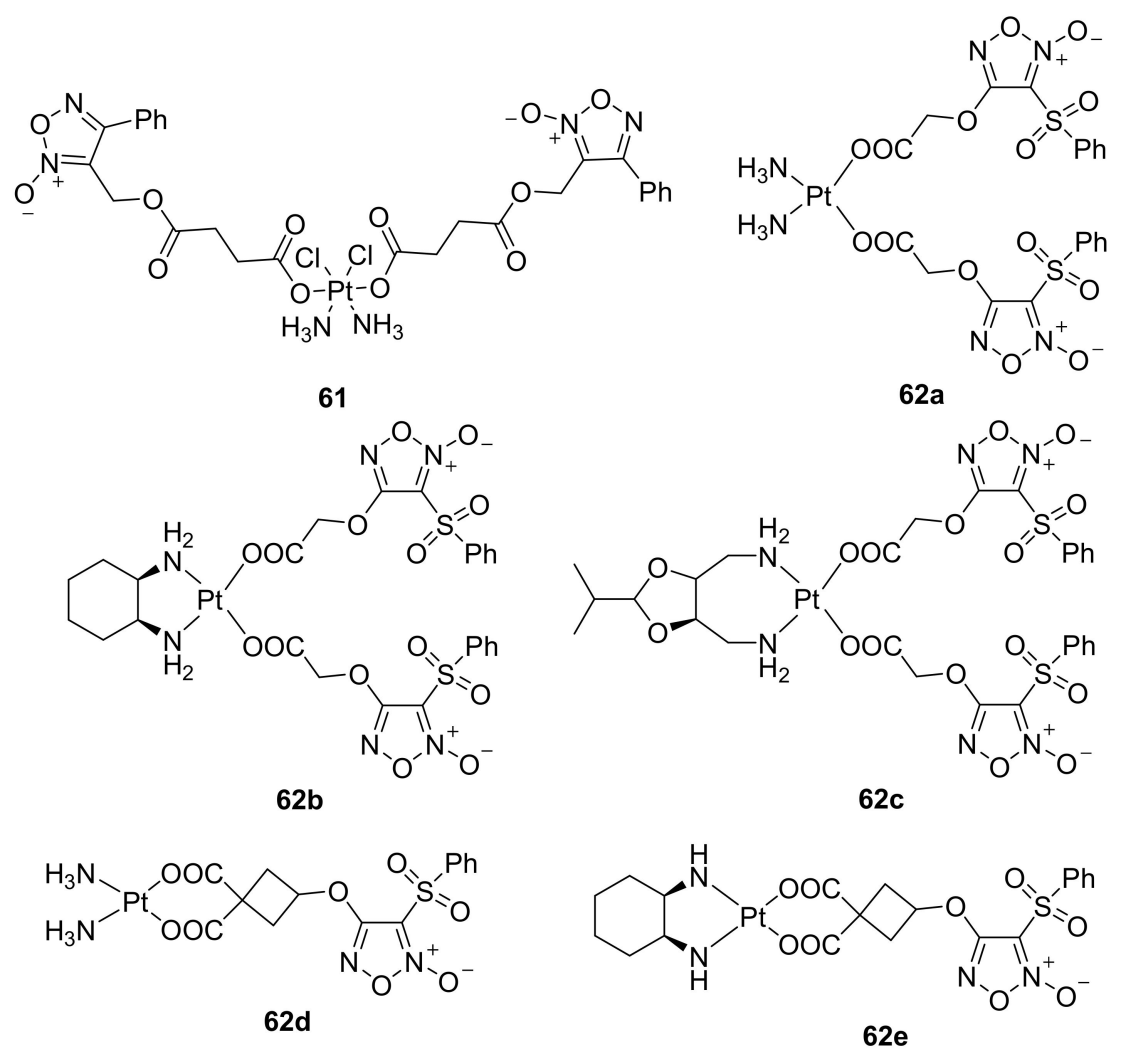
Anticancer furoxan-based Pt-complexes.

A series of anticancer Pt complexes bearing a NO-donor furoxan subunit was synthesized (Figure 5). Compound **61** is a cisplatin prodrug, which releases it directly in cells along with NO. Such a dual role of hybrid **61** provides a synergistic effect on the inhibition of tumor growth which is demonstrated both by higher potency and lower systemic toxicity in comparison with parent cisplatin. Pt–furoxan derivatives did not alter the growth of mice in both dosages, suggesting the low toxicity of drug candidates. By comparison, cisplatin clearly decreased the bodyweight of mice, and even one mouse died during the treatment of cisplatin (1–2 mg/kg dosages were used) [104]. Other Pt-organic complexes may serve as a potential replacement for cisplatin and oxoplatin. The anti-cancer effect of **62a–e** was attributed to their interaction with DNA, which formed Pt/DNA adducts responsible for cytotoxicity [105].

**Scheme 32:**
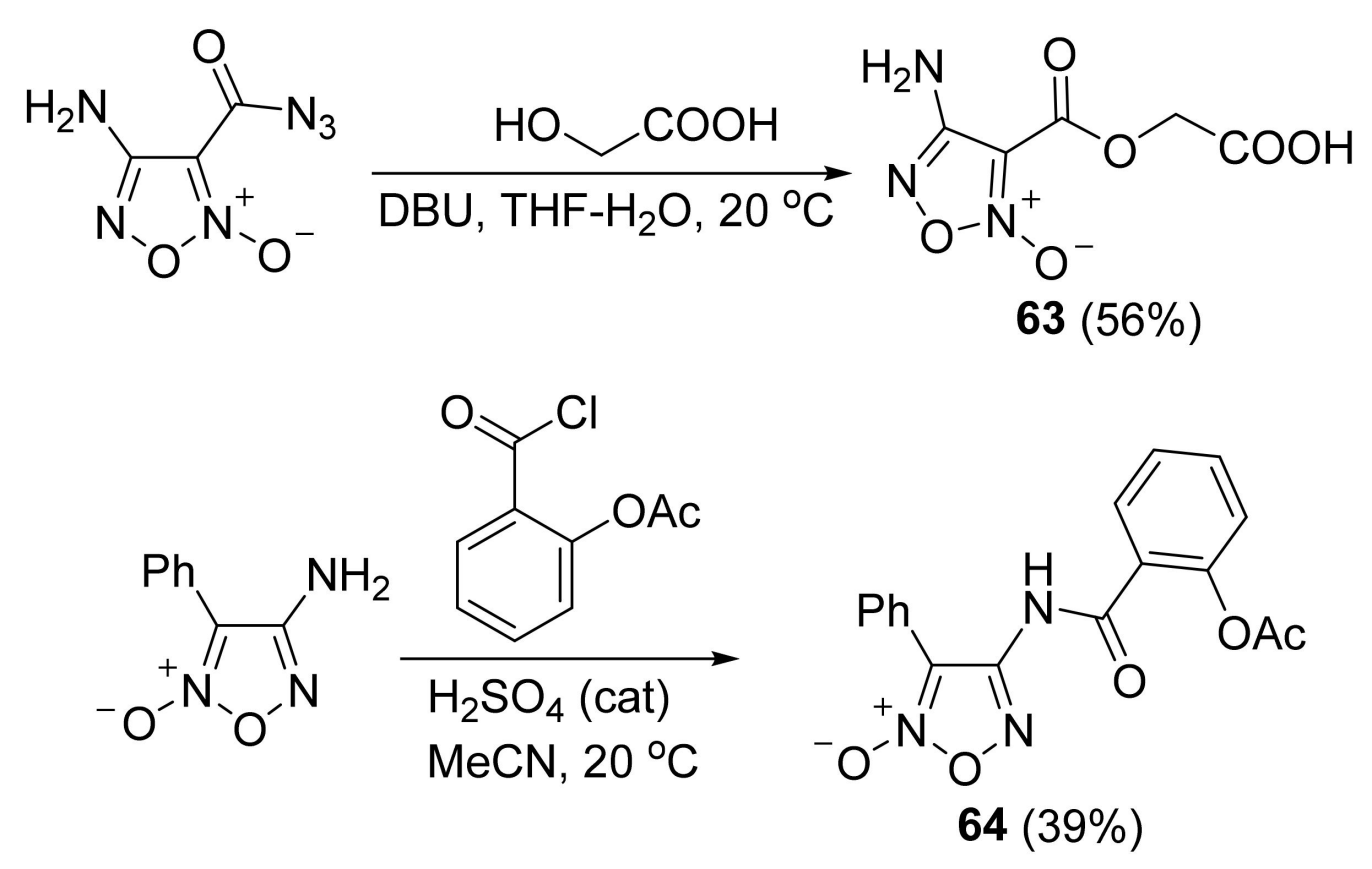
Synthesis of furoxan-based antiplatelet agents.

Hybrid structures **63** and **64** comprising of the furoxan motif and glycolic or acetylsalicylic (aspirin) acids scaffolds were synthesized (Scheme 32). Both compounds revealed high antiplatelet activity which was comparable to that of the previously investigated CAS 1609. In addition, these structures possess a selective mechanism of inhibition of platelets aggregation mediated by ADP and adrenaline but not ristocetin, collagen and arachidonic acid and their antiplatelet effects are mainly independent of the NO action [106].

**Scheme 33:**
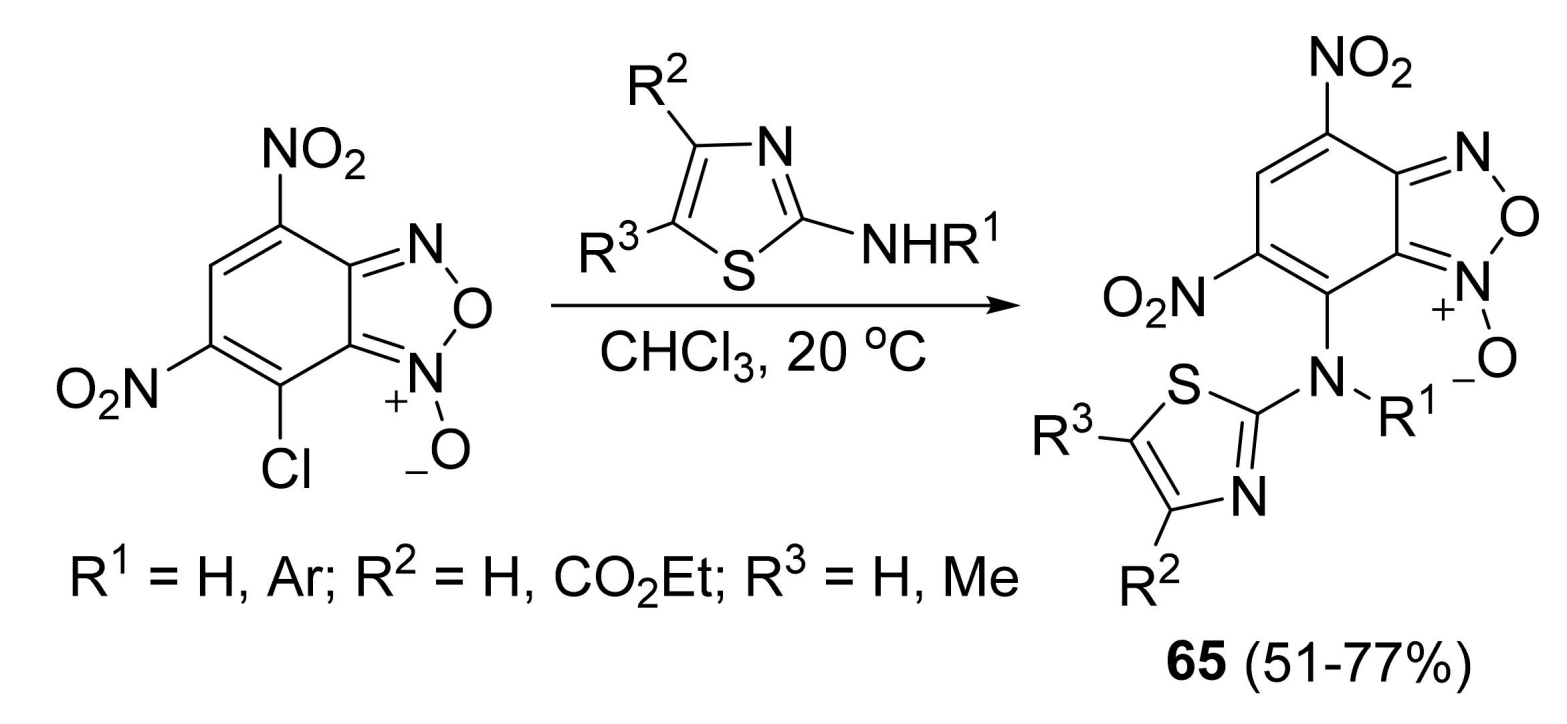
Synthesis of benzofuroxan–aminothiazole hybrids.

Several approaches to the functionalization of pharmacologically relevant benzofuroxans were also developed. Coupling of benzofuroxancarboxylic acid with pharmacophoric amines afforded a series of corresponding amides, in which a thiomorpholine derivative revealed an excellent antibacterial activity against drug-resistant strains of *Mycobacterium tuberculosis* [107]. Tri- and tetracyclic ring systems incorporating a nitrofuroxanoquinoline scaffold revealed extraordinary high NO-donor ability which may be useful for a search of novel drug candidates in this series [108]. Benzofuroxans bearing additional electron-withdrawing groups are also considered as super-electrophiles which is convenient for rapid and straightforward installation of various pharmacophoric moieties to the benzofuroxan core. Recently, a thorough investigation of the reactivity of 4,6-dichloro-5-nitrobenzofuroxan with aliphatic and aromatic amines was performed [109]. Using a super-electrophilic nature of the benzofuroxan ring system, a series of promising anticancer drug candidates **65** bearing 2-aminothiazole moiety was synthesized (Scheme 33). The cytotoxic activity of the lead benzofuroxan derivative bearing 4-methoxyphenyl substituent at the aliphatic nitrogen atom was attributed to induction of apoptosis in m-HeLa cells at early stages (10.7–17.3% of apoptotic cells were observed using 50–100 μM concentrations) [110].

A number of other furoxan derivatives also displayed a wide range of pharmacological activities. For example, 3-cyano- and 3-nitro-4-phenylfuroxans revealed promising antiplatelet activity [111], while the latter compound also inhibited *Pseudomonas aeruginosa* PAO1 growth and prevented PAO1 biofilm formation [112]. Several furoxan–peptide hybrids also demonstrated antimicrobial activity against *Staphylococcus aureus* and *Escherichia coli* biofilm formation [113]. A highly potent drug candidate PRG150 (3-methylfuroxan-4-carbaldehyde) is considered as a novel analgesic for the effective treatment of painful diabetic neuropathy. PRG150 is useful for relieving mechanical allodynia, a defining symptom of neuropathic pain, as was shown in a rat model of painful diabetic neuropathy [114]. The metabolic stability and biodistribution of PRG150 using positron emission tomography and ^11^C-and ^13^N-labeled pharmaceutical demonstrated a higher uptake of the ^13^N isotope over ^11^C in the spinal cord, which indicates a key role of NO release in the somatosensory nervous system responsible for the analgesic effect of PRG150 [115]. Subsequent in-depth studies confirmed a role for NO in the treatment of painful diabetic neuropathy since the cellular effects of PRG150 on forskolin-stimulated cyclic adenosine monophosphate (cAMP) responses in vitro and in vivo were transduced through the modulation of μ-opioid receptor signaling [116]. Recently, a biomedical application of diacylfuroxans as precursors to nitrile oxides capable of covalent inhibition of glutathione peroxidase 4 (GPX4) to induce ferroptosis was revealed [117]. Application of furoxan-based compounds as high-energy materials, primary or secondary explosives, propellants or rocket fuels was also reported numerously [118,119,120,121,122].

## 4. Pyridazine Dioxides and Azasydnones

The final chapter of the present review covers several aspects of the synthesis and biomedical applications of two rather neglected heterocyclic subclasses: pyridazine dioxides and azasydnones (1,2,3,4-oxatriazolium-5-olates). Historically, both these heterocyclic derivatives are known as exogenous NO-donors for more than 30 years [27]. However, substrate-specific and harsh methods for their synthesis significantly restricted in-depth investigation of their functional properties. Nevertheless, recent achievements of several research groups provided some solutions to this problem and modern convenient synthetic protocols to an assembly of both pyridazine dioxide and azasydnone frameworks along with their application potential are summarized in this section.

**Scheme 34:**
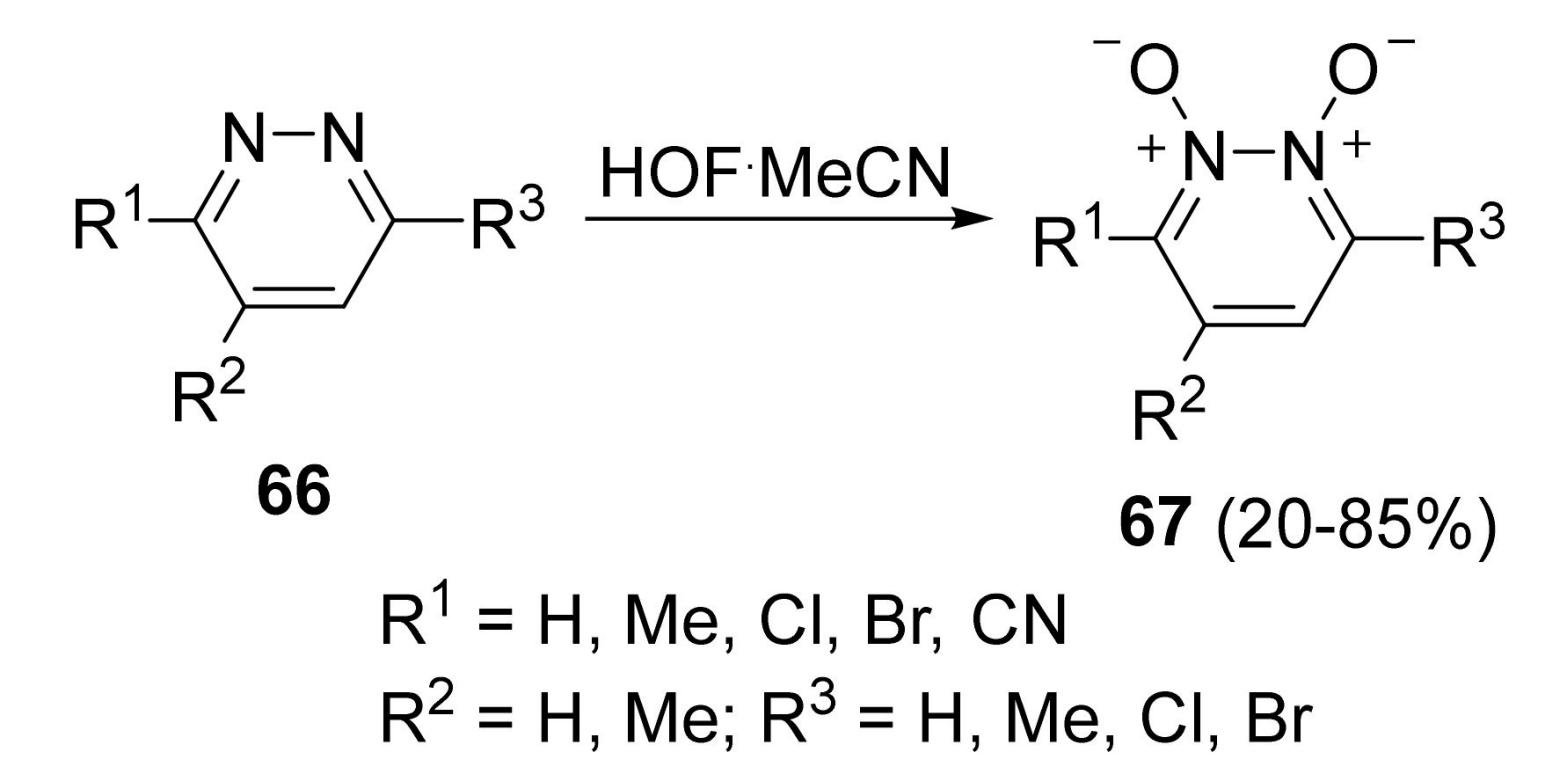
Direct oxidation of pyridazines.

Direct oxidation of pyridazines **66** with peracetic or pertrifluoroacetic acid proceeds with poor yields of the corresponding dioxides **67** [123]. Recently, a more effective oxidation method using one of the strongest oxidizers HOF was developed (Scheme 34). However, significant amounts of pyridazine mono-*N*-oxides were also formed [124].

**Scheme 35:**
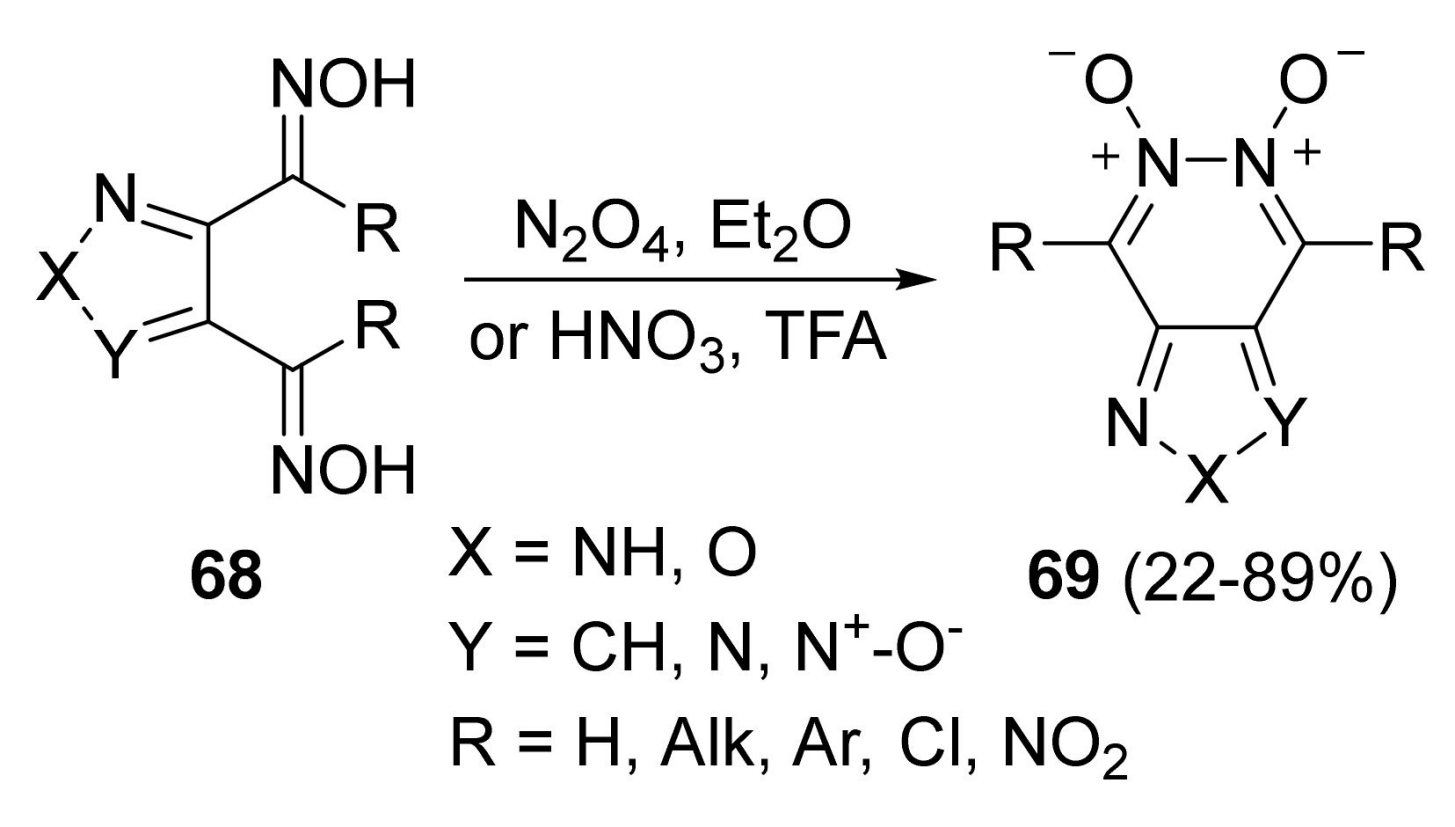
Oxidative cyclization of 1,4-dioximes.

A more convenient approach toward the formation of the pyridazine dioxide scaffold is based on an oxidative cyclization of 1,4-dioximes **68**. This method is more efficient and provides high yields of target heterocyclic NO-donors **69** including those comprised of a pyridazine dioxide motif fused with other heterocyclic rings (pyrazole [125], furazan [126], furoxan [127,128]) (Scheme 35). Initially, PhI(OAc)_2_ [129] or Pb(OAc)_4_ [130,131] were used as oxidants, but later N_2_O_4_ [125,126,128] or HNO_3_ [127] were shown to provide higher yields and purity of pyridazine dioxides **69**.

Aryl substituted 2*H*-pyrazolo [3,4-*d*]pyridazine 5,6-dioxides produced low amounts of NO (4.2–10.5%), while those bicyclic derivatives comprising of NO-donor furoxan and pyridazine dioxide subunits demonstrated the highest NO-releasing ability (27.7–48.3%) [125]. Furthermore, 4,7-dimethyl-1,2,5-oxadiazolo[3,4-*d*]pyridazine 1,5,6-trioxide was able to relax noradrenaline-precontracted aortic rings at concentrations less than 0.1 mM. Deoxygenated furazan analog was less active since the furazan subunit is unable to release NO. Nevertheless, both these compounds significantly increased cGMP levels in aortic rings and platelets and exhibited promising antiaggregant activity [132]. Unfortunately, the mechanism of NO release from pyridazine 5,6-dioxides is unknown so far.

**Scheme 36:**
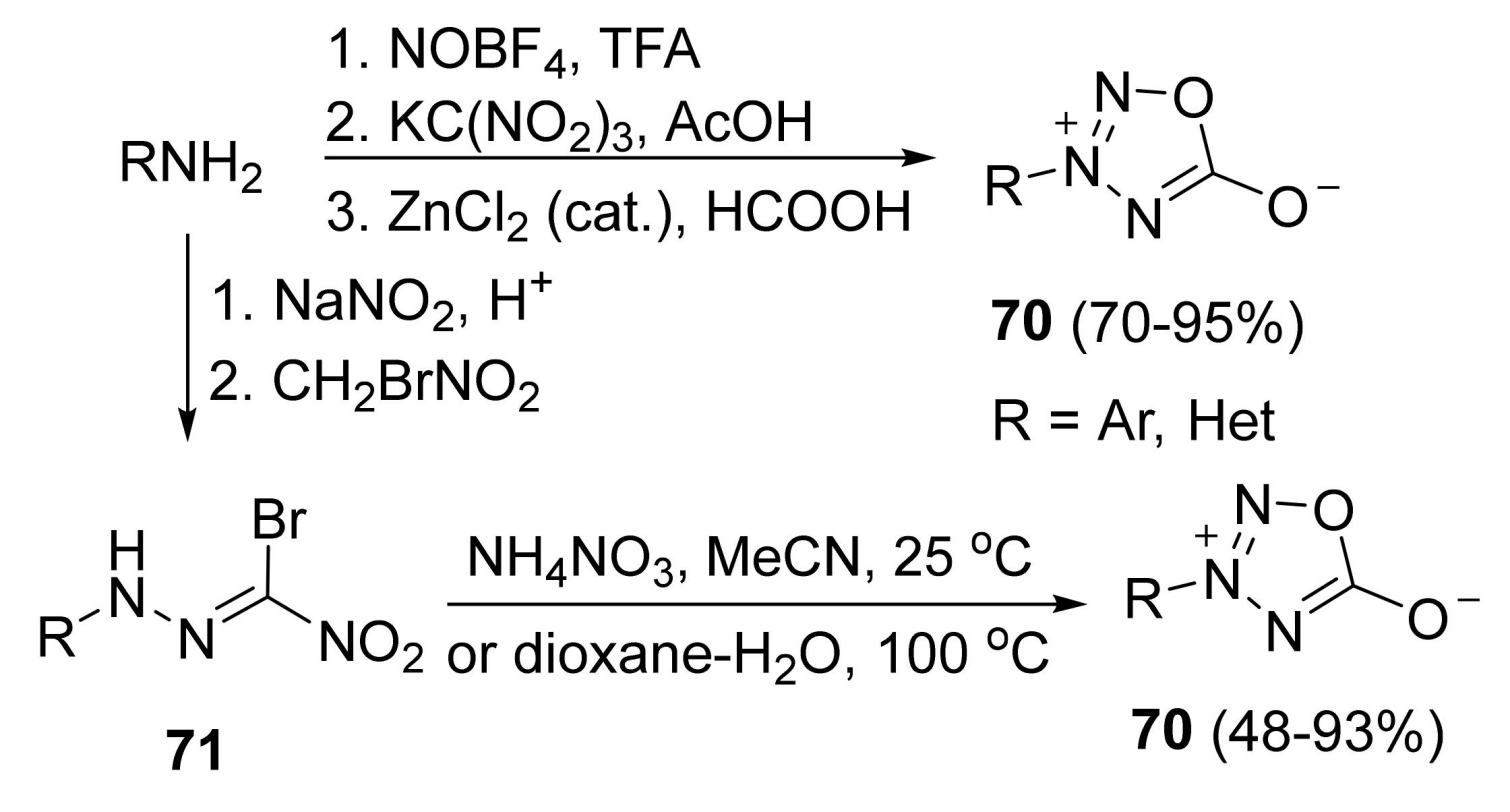
Synthesis of azasydnones.

Azasydnones **70** are usually constructed by azo coupling of tailor-made or generated in situ arene/hetarene diazonium salts with bromonitromethane with subsequent oxidative [133] or thermal [36] cyclization of the corresponding hydrazones **71**. Recently, our group proposed a more convenient one-pot approach using potassium nitroformate and catalytic amounts of ZnCl_2_ which enabled a straightforward assembly of the azasydnone ring (Scheme 36) [134].

Due to pronounced NO-releasing properties, azasydnones possess promising antihypertensive activity and low toxicity [135,136]. In addition, (1,2,5-oxadiazolyl)azasydnones showed excellent antiplatelet activity in the case of ADP and adrenaline used as inducers completely suppressing the formation of the aggregates, which is rather a unique feature. Importantly, (1,2,5-oxadiazolyl)azasydnones possess a selective mechanism of inhibition of platelets aggregation mediated only by ADP and adrenaline, which are considered to be the main agents causing thrombus formation [137]. Additionally, recently, some energetic applications of azasydnones were reported [138,139,140,141].

**Scheme 37:**
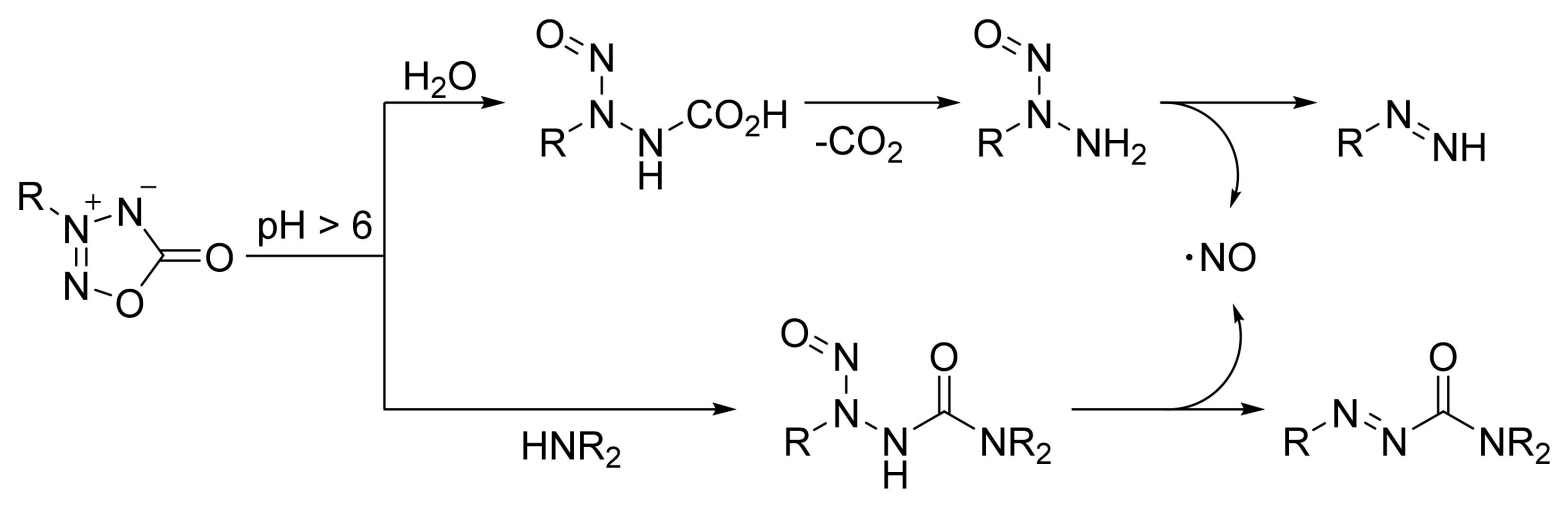
Mechanism of NO release from azasydnones.

Mechanistic investigations on NO-donating properties of azasydnones revealed their ability to release NO at pH > 6 either in vitro or enzymatically. This process includes nucleophilic cleavage of the azasydnone ring followed by stepwise degradation of formed nitrosamines (Scheme 37). Degradation of (1,2,5-oxadiazolyl)azasydnones afforded benzoic acid as a main decomposition product due to the concomitant cleavage of the 1,2,5-oxadiazole ring. Since benzoic acid is also non-toxic for living organisms, (1,2,5-oxadiazolyl)azasydnones are considered advantageous in future drug design [137].

## 5. Conclusions and Future Outlooks

Synthesis and reactivity of heterocyclic NO-donors have become one of the urgent areas of research in organic and medicinal chemistry. In comparison to clinically used organic nitrates, heterocyclic scaffolds capable of NO release under physiological conditions are more advantageous due to hydrolytic stability and improved pharmacological profiles. Aside from studying biomedical applications of simple heterocyclic NO-donors, numerous efforts were directed towards the construction of hybrid pharmaceuticals incorporating NO-donor heterocyclic subunits as a key structural fragment to advance the pharmacological profile of the parent drug. To realize the hybridization approach, a number of modern synthetic methodologies involving preliminary functionalization of the heterocyclic ring with subsequent hybridization with an appropriate pharmacophore were utilized. This approach is very promising for the construction of novel multifunctional drugs to overcome the significant problem of multidrug resistance. In general, diversification of the heterocyclic NO-donor scaffold has become a reliable tool in modern organic chemistry; therefore, the creation of novel pharmacologically active lead hybrids with NO-donor properties should be expected in near future.

In this review, recent advances in the synthesis, reactivity and pharmacological activity of the main NO-donor heterocyclic subclasses are summarized. Although the structures of considered nitrogen–oxygen molecular systems are quite similar, synthetic methods for their preparation and functionalization differ significantly. The synthesis of sydnone imines and furoxans was extensively studied and nowadays main trends are directed toward selective functionalization of these heterocycles. Furthermore, incorporation of the sydnone imine or furoxan motif in the structure of known pharmaceutical or promising drug candidates was found to be fruitful in search of novel pharmacologically active and non-toxic substances. In addition, sydnone imines are highly valuable substrates for bioorthogonal chemical reactions, which are of major importance in the field of chemical biology. On the contrary, the chemistry of pyridazine dioxides and azasydnones is explored to a lesser extent since both these heterocyclic subunits were hard to construct. Nevertheless, recently developed novel and convenient methods for the assembly of pyridazine dioxides and azasydnones will encourage further investigations on the pharmacological activity of these compounds. Overall, each type of considered heterocyclic NO-donors has a strong potential in medicinal chemistry and drug design and we hope that the present review will stimulate future research in this field.
